# Multicomponent Analysis of the Differential Induction of Secondary Metabolite Profiles in Fungal Endophytes

**DOI:** 10.3390/molecules21020234

**Published:** 2016-02-18

**Authors:** Víctor González-Menéndez, Mercedes Pérez-Bonilla, Ignacio Pérez-Victoria, Jesús Martín, Francisca Muñoz, Fernando Reyes, José R. Tormo, Olga Genilloud

**Affiliations:** Fundación MEDINA, Parque Tecnológico Ciencias de la Salud. Avda. del Conocimiento 34, 18016 Granada, Spain; mercedes.perez@medinaandalucia.es (M.P.B.); ignacio.perez-victoria@medinaandalucia.es (I.P.V.); jesus.martin@medinaandalucia.es (J.M.); francisca.munoz@medinaandalucia.es (F.M.); Fernando.reyes@medinaandalucia.es (F.R.); jose.tormo@medinaandalucia.es (J.R.T.)

**Keywords:** fungal endophytes, epigenetic modifiers, HDAC, DNMT, volcano plots, secondary metabolites, metabolomics

## Abstract

Small molecule histone deacetylase (HDAC) and DNA methyltransferase (DNMT) inhibitors are commonly used to perturb the production of fungal metabolites leading to the induction of the expression of silent biosynthetic pathways. Several reports have described the variable effects observed in natural product profiles in fungi treated with HDAC and DNMT inhibitors, such as enhanced chemical diversity and/or the induction of new molecules previously unknown to be produced by the strain. Fungal endophytes are known to produce a wide variety of secondary metabolites (SMs) involved in their adaptation and survival within higher plants. The plant-microbe interaction may influence the expression of some biosynthetic pathways, otherwise cryptic in these fungi when grown *in vitro*. The aim of this study was to setup a systematic approach to evaluate and identify the possible effects of HDAC and DNMT inhibitors on the metabolic profiles of wild type fungal endophytes, including the chemical identification and characterization of the most significant SMs induced by these epigenetic modifiers.

## 1. Introduction

Arid zones in Andalucía have special edaphological and climatic conditions such as low rainfall, high sunshine levels and specific lithology where loamy materials and evaporites abound. Native plant communities from these arid zones possess distinctive survival characteristics in these special conditions, which have led to the existence of a high degree of endemic plants with highly adapted endophyte ecosystems poorly studied. It is precisely this singularity which turns them into a valuable potential source for the isolation of new unique host-specific endophytes.

Fungal endophytes consist mainly of members of the *Ascomycota* class of mitosporic fungi, but can also include members of the *Basidiomycota*, *Zygomycota* and *Oomycota* classes [1]. Some of these groups are widespread and found on different plant species; others are highly specific to single hosts and single environments. Fungal endophytes are organisms inhabiting plants which are characterized by being neutral or beneficial to the host plant. These fungi can promote plant growth and confer abiotic and biotic stress tolerances throughout one or more interactions with their host, for example, by producing a wide variety of other secondary metabolites (SMs). These interactions may influence the expression of some biosynthetic pathways, otherwise cryptic when these fungi are grown *in vitro* in laboratory conditions.

**Figure 1:**
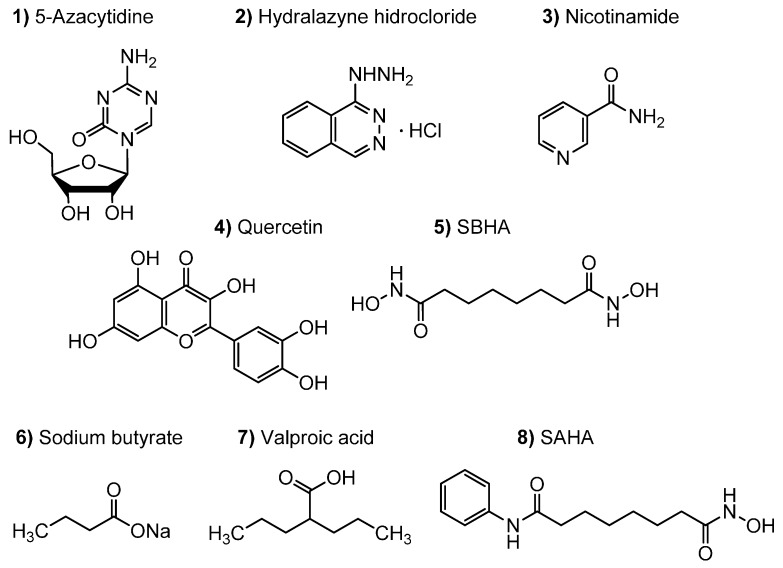
Epigenetic small-molecule modifiers **1**–**2** of DNA methyltransferase and **3**–**8** of histone-deacetylase activities described in the study.

Epigenetic small-molecule modifiers (Figure 1) of DNA methyltransferase (DNMT) and histone deacetylase (HDAC) activities are being used to perturb the fungal secondary biosynthetic mechanisms, which can lead to the induction of the expression of silent metabolite pathways. DNMT inhibitors such as 5-azacitidine (**1**) and hydralazine hydrochloride (**2**) have demonstrated their ability to reduce DNA-methylation-mediated silencing of different resistance genes and cellular processes in a wide variety of fungal species [2].

Three classes of histone deacetylases (HDAC) (I, II and III) have been identified in fungi [3]. Sirtuins are the most described ones (HDAC of class III). They are NAD-dependent protein deacetylases involved in a wide variety of biological processes, including transcriptional silencing, regulation of apoptosis, fat mobilization, and stress resistance [4]. Sirtuins’ activities are regulated by nicotinamide (**3**), a noncompetitive inhibitor that promotes a base-exchange reaction at the expense of deacetylation [5], and quercetin (**4**), a natural product belonging to the flavonoids family, that acts as an activator of sirtuins activities [6]. Quercetin has antioxidant properties, modulates the expression of specific enzymes attributed to an inhibitory action on protein kinases, inhibits DNA topoisomerases and regulates gene expression [7]. Recent examples on the use of nicotinamide (HDAC of class III inhibitor) can be the induction in *Chaetomium cancroideum* of the secondary metabolites chaetophenol G and cancrolides A and B [8].

Suberohydroxamic acid (**5**), sodium butyrate (**6**) and valproic acid (**7**) are potent inhibitors of HDAC of classes I and II. Several reports have described the variable effects of HDAC inhibitors on fungi. The majority of these epigenetic studies report the effect of using hydroxamic-acid compounds, such as SAHA (**8**), with similar HDAC inhibitory effects; e.g.,: the production of a new fungal biosynthetic cyclodepsipeptide (EMG-556) by *Microascus* sp. when cultured with SAHA [9], the production of new anti-infective cytosporones by the marine fungus *Leucostoma persoonii* [10] or, the biosynthesis of novel fusaric acid derivatives by the *Datura stramonium* endophyte when SBHA was added to its production medium [11].

Recently Albright *et al.* [12] proved global changes in *Aspergillus nidulans* metabolome upon treatment with epigenetic modifiers in response to an upregulated reduced expression of rpdA by using volcano-plots statistical analyses [13]. This study suggested that, although HDAC inhibition generally leads to an up-regulation of the *Aspergillus nidulans* biosynthetic machinery as evidenced by transcriptomics, the response at the level of the secondary metabolome is more complex than just a global increase in the abundance of the secondary metabolites already produced by the strain.

The principal aim of this study was to evaluate the differential expression of biosynthetic pathways of wild fungal endophytes upon systematic treatment with different small molecule elicitors. The multicomponent analysis of the differential induction of secondary metabolite profiles by applying metabolomic techniques may result in a robust methodology for assessing the potential effects of HDAC and DNMT epigenetic modifiers on the secondary metabolites profiles of fungal endophytes of interest, and also, promoting systematically the expression of their cryptic pathways typically silent when fermented *in vitro*.

## 2. Results and Discussion

### 2.1. Fungal Isolation and Identification

The collection areas were localized in arid regions of the provinces of Granada and Almeria (Andalucía, Spain). Twelve different species of representative plants characteristic of each geographic region were collected. The geographical origins and isolation substrata of the strains are listed in Table 1. For the taxonomic determination of their genus and species, sequencing of the complete ITS1-5.8S-ITS2-28S region or independent ITS and 28S rDNA were performed and compared with GenBank and the NITE Biological Resource Center (http://www.nbrc.nite.go.jp/) databases by using the BLAST application [14].

Thirteen different fungal strains were finally isolated and identified from these plants, that can be grouped into two taxonomic categories: (i) a taxonomically homogeneous group consisting of seven different species of the *Dothideaceae* family, and (ii) an heterogeneous group made up of six endophytes belonging to different fungal families (*Chaetothyriaceae*, *Phaeosphaeriaceae*, *Planistromellaceae*, *Sporormiaceae* and *Xylariaceae*, Table 1).

### 2.2. Screening of Epigenetic Modifiers Effects on Fungal Endophytes

All strains were grown in the same production medium (YES) in the absence (control) and presence (100 μM) of seven small-molecule epigenetic modifiers that were added: (i) only during the production stage (− +), or (ii) during both, seed and production, fermentation stages (+ +). According to previous reports [10,15], all epigenetic modifiers were tested at 100 μM as the standard concentration in the study. After 14 days of fermentation, ensuring the generation of enough biomass in the control conditions, submerged culture differences in morphology and SMs profiles were analyzed visually and by UHPLC-UV at 210 nm. Changes in production titers (as changes in 210 nm UV areas) and presence of new peaks were observed among the different treatments with respect to their corresponding controls. Increasing changes in production profiles were classified into three categories as ***p-pp-ppp*** (as increasing changes: ×2, ×4 and ×8 respectively, in the area of the peaks under the uHPLC UV-210 nm trace compared to its corresponding control areas). Changes in the chemical diversity induced were also categorized based on the number of new UHPLC-UV 210 nm peaks detected as ***d***: 1–3 peaks; ***dd***: 3–5 peaks and ***ddd*** > 5 peaks (Table 2). In addition, any changes in the fermentation morphology (pigmentation, final biomass or conidia/hyphae conversion rate), were also referred as ***m*** in the table.

**Figure 2:**
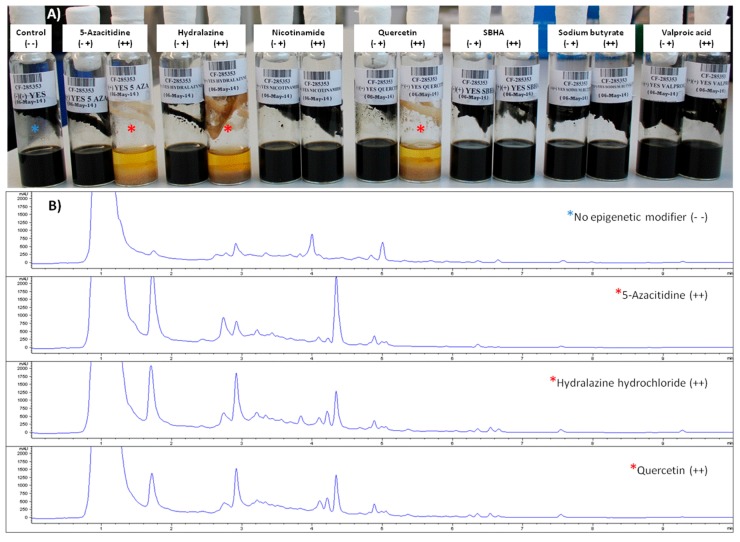
(**A**) Fungal strain CF-285353 grown, for 14 days in YES medium with and without all the epigenetic modifiers studied, showing differences in texture, colour, morphology and biomass amount; (**B**) Comparative analysis of the differential UHPLC-UV 210 nm secondary metabolite profiles produced by the strain with and without the addition, during seed and the production steps (+ +), of the three epigenetic modifiers of interest (red asterisks) *vs*. control (blue asterisk).

Three of the 13 fungal strains screened by using the 14 epigenetic modifiers fermentation conditions showed: clear significative changes in their final growth morphologies, changes in their production titers and/or generation of new peaks when cultured in the presence of the epigenetic modifiers. Among them, the strain that showed more changes in these three components was the strain *Dothiora* sp. CF-285353. This strain was therefore selected for a deeper and more detailed metabolomic study on the effects of the three elicitors that induced most morphological and SMs profile changes when compared to its corresponding control fermentation condition (Figure 2).

### 2.3. Standardization of Cultivation Conditions of Dothiora sp.

The study on the effect of the epigenetic modifiers has shown that the two DNMT inhibitors, 5-azacitydine (**1**) and hydralazine hydrochloride (**2**), and the sirtuin activator quercetin (**4**), induced more changes in the SMs profiles of the strain CF-285353 when added both, to the seed and production stages (+ +), than when added only during the production stage (− +). To verify that these changes were significative and reproducible, extensive studies were designed and carried out with submerged cultures per triplicate, in the presence and absence of these three elicitors, both in the seed and/or the production stages.

The cultivated strain CF-285353 presented different morphologies after addition of some of the epigenetic modifiers (Figure 2). To ensure the reproducibility of the experimental conditions, we characterized the morphology of the strain both in the inoculum and in the different fermentation steps. We considered, as well, the risk of introducing mutations and strain degeneration after each transfer and cell division by the liquid-liquid sub-culturing required to obtain enough inoculum for the study [16]. This effect has been previously observed in studies comparing jasmonic acid production by a fresh and a sub-cultured producing strain, which concluded that liquid-liquid sub-culturing led to a significative downfall of jasmonic acid production by *Lasiodiplodia theobromae* [17].

**Figure 3:**
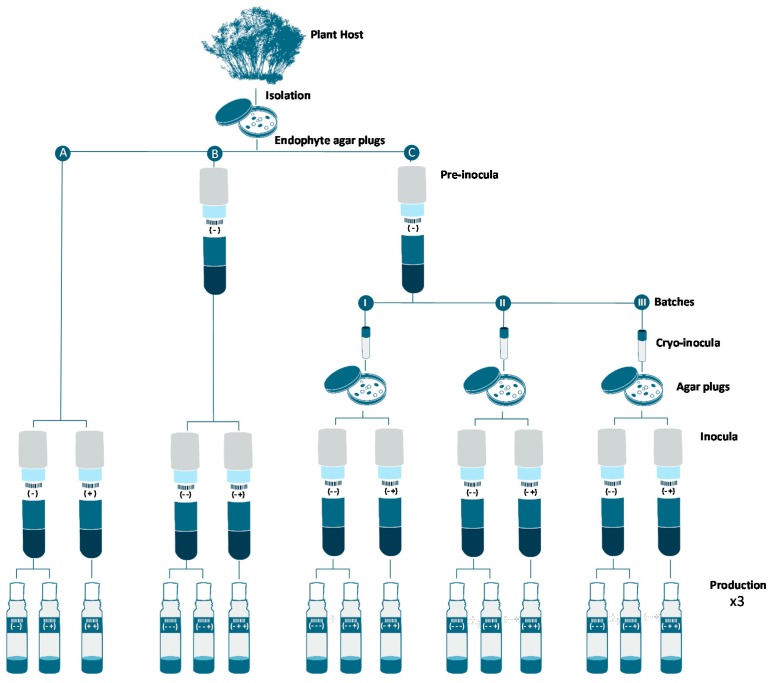
Experimental design for evaluating the effect of sub-culturing and epigenetic modifiers in *Dothiora* sp. (**A**) Initial fungal fermentation process; (**B**) Pre-inocula step added; (**C**) Pre-inocula and agar plugs steps added.

Therefore, three experimental designs where tested for minimizing these inoculum sub-culturing effects: (i) a method involving the use of one single stage seeds from agar plugs, similar to the original process performed during the previous screening of the epigenetic modifier additives (Figure 3A); (ii) a second method where a pre-inoculum enrichment step was added for generating a large amount of inoculum from a unique production batch (Figure 3B); and (iii) a third one involving a pre-inoculum, preserved as frozen stocks, from which agar plates of the endophyte with a controlled differentiation morphology were prepared, and were later used to inoculate seeds for each production batch (Figure 3C).

Finally, and similarly to the screening procedure, each inoculum generated was then seeded in the production medium (YES), in absence (control) and presence (100 μM) of the three best small-molecule epigenetic modifiers selected: (i) added only during production or; (ii) added during all fermentation steps, seed and production.

#### 2.3.1. Effects of Epigenetic Modifiers on *Dothiora* sp. Submerged Culturing

**Figure 4:**
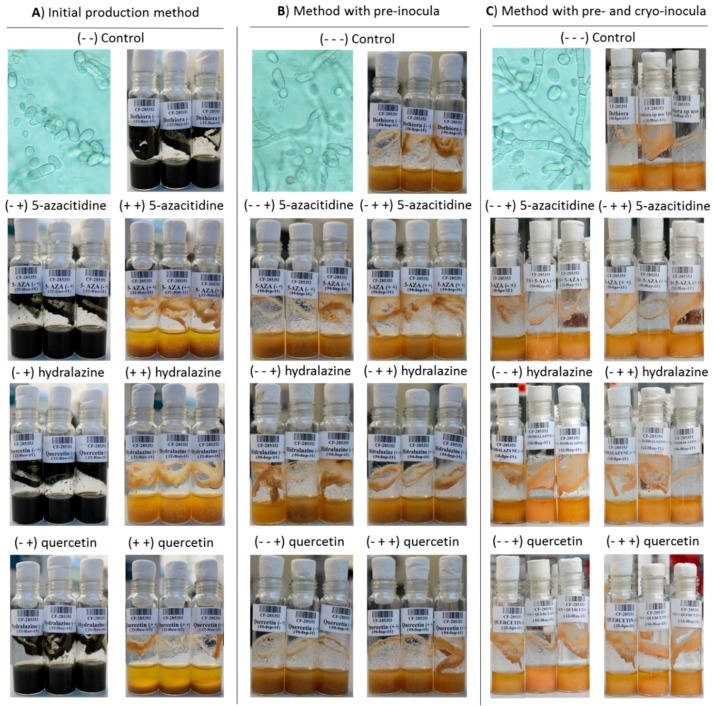
Fermentation batches obtained for: the initial production method (**A**); when a pre-inocula step was added (**B**); and when a pre-inocula and agar-plug steps were added (**C**).

To assess the effect of the epigenetic modifiers, all fungal cultures were harvested after 14 days of incubation, and were characterized according to their morphology (color, biomass and mycelial-to-yeast conversion phase; Figure 4), extracted with organic solvent and analyzed by UHPLC-MS.

Regarding the morphology, clear differences were observed between the three control fermentation batches for each sub-culturing methods tested even without any epigenetic modifier. The fermentation triplicates performed using the initial screening approach (Figure 3A) presented, consistently, black thick broth with a high density of conidia (Figure 4A), whereas fermentation controls for the other two inoculating methods (Figure 3B,C) resulted in a yellowish broth with plenty of hyphae and few conidia (Figure 4B,C). A slightly higher amount of hyphae was present when using the frozen inocula (Figure 3C), even when harvested after at 7 days, half of the time used for the other two methods (14 days). No other morphology differences could be related to the presence or absence of any of the epigenetic modifiers, thus all three methods and the two growth morphologies were decided to be included in further metabolomic differential analyses.

#### 2.3.2. Metabolomic Evaluation of the Changes in the SM Profile of *Dothiora* sp. by Epigenetic Modifiers

HPLC-UV traces can be used for evaluating the effect of epigenetic modifiers on fungal strains and have proved to be suitable for a fast detection of the conditions that induce strong changes in their SM profiles. In our case, extended studies required deeper analytical methods to quantify and identify the SMs that could be induced by the addition of the modifiers. We decided to use mass spectrometry analyses, as metabolomics is currently getting stronger foundation on LC/MS data. In recent studies, Volcano-plots have been applied to these data with success in describing and obtaining conclusions on the biosynthesis of fellutamides by *A. nidulans*, hence supporting the use of HDAC inhibitors and said techniques for the discovery of cryptic secondary metabolites [12,13].

Volcano-plots constitute a scatter-plots representation that can describe very visually how two different experimental conditions may affect a large set of components. Statistically, these plots determine if significative differences exist between averages of two populations of the same component treated with the two conditions of interest, depicting these results for, in our case, the secondary metabolites of a given extract (or the mass ions that can be detected by LCMS and can be inferred as components).

**Figure 5:**
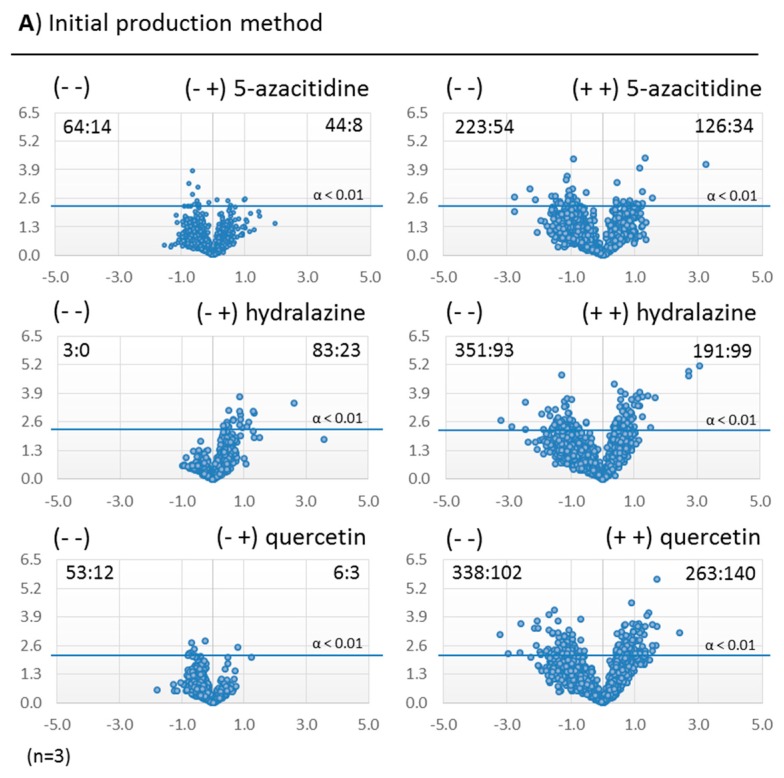

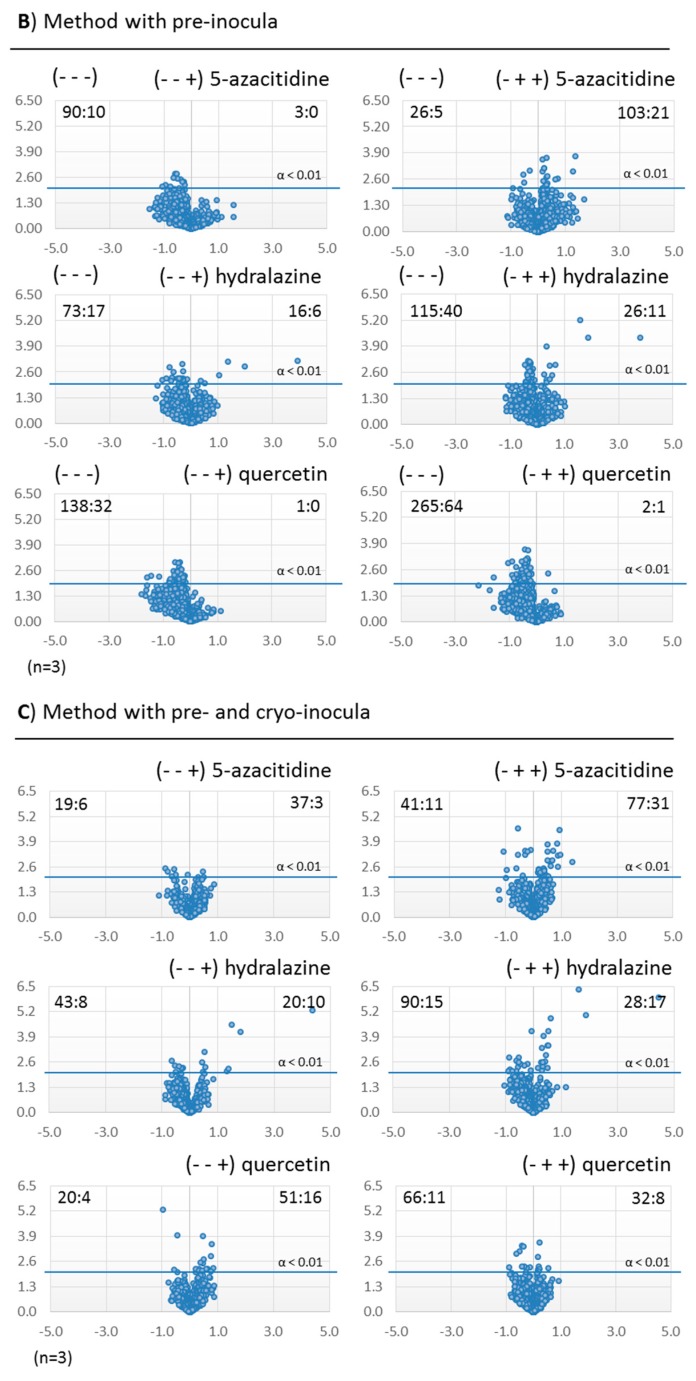
Volcano-plots for the three production methods tested, comparing the different growth conditions tested with their corresponding controls (−log 10 of *t*-test statistical *p*-value in *y*-axis *vs.* −log 2 of ion masses areas ratio in *x*-axes): (**A**) Initial production method; (**B**) pre-inoculum step added; (**C**) pre-inoculum and agar-plug steps added. The number of statistically different mass ions due to higher production for each growth condition is indicated as n_1_:n_2_, where n_1_ and n_2_ are respectively the number of differential mass ions with statistical confidences of 95% and 99% (*n* = 3; α < 0.05 and α < 0.01 respectively; 99% of significance (α < 0.01 limit) depicted).

In *Dothiora* sp. fermentations (Figure 3 and Figure 4), we observed molecular species whose production increased or decreased significantly with respect to the corresponding controls for each condition (see Figure 5 for a detailed number of the ions statistically different in production for each modifier and fermentation method). In general, the addition of small-molecule elicitors, both during the inocula and the production fermentations, resulted in a significant increase of the diversity and amount of secondary metabolites generated for the three epigenetic modifiers of the study. For the two seeding methods that have included a pre-inoculum step, 5-azacytidine (**1**) was the epigenetic modifier that induced more changes when added both during the seed and production fermentations, and the number of significant unique molecular species detected by HPLC-MS at least doubled when compared to its addition only during the production fermentation.

In most of the cases we observed a continuous dispersion of the *p*-values for the significance of the differential components for confidences above 95% and 99%. This indicated a general and continuous epigenetic modifiers effect on the SMs profiles, without highlighting any outlier group of ions that could indicate a unique strongly induced secondary metabolite pathway. Thus, results confirmed the general non-specificity of the effects induced by the modifiers on the culture broths SMs profiles. In fact, only hydralazine was observed to induce clear groups of outlier mass ions in the scatter-plots.

The conditions showing less production changes on the SMs abundances compared to their controls were the fermentations that were prepared with the pre- and cryo-inocula method (Figure 5C). In contrast, the higher dispersions were obtained with the initial production method (Figure 5A). Although fermentations were harvested with similar biomasses, the method that included pre- and cryo-inocula steps presented the lowest dispersion of their volcano-plots (Figure 5C), suggesting an important influence of the methodology used.

#### 2.3.3. Identification of Molecules Produced in the Fermentations with Modifiers

As previously commented, hydralazine (**2**) was the epigenetic modifier that induced most of the differential mass ion populations for CF-285353. In fact a total number of 23 and 99, 6 and 11, and 10 and 17 ions were identified respectively as statistically differential, with a 99% of significance, for treatments with hydralazine in (− +) and (+ +) for methods A, B and C (see Figure 5 for details). Among them, we could identify some molecules with 8 to 32 fold higher production rates in the presence of this modifier, and some others that were only produced in its presence.

In an initial attempt to identify several of the natural products induced by this modifier, we compared every ion that presented a statistical significance above 99% to our internal de-replication UV-HPLC-HRMS databases. Database matching was performed by using an in house developed application where the UV signal, retention time, mass signal and moleculat formula of the selected ions are compared to the UV-HPLC-HRMS data of known metabolites stored in our proprietary database of 845 microbial natural products, including commercial compounds and molecules obtained from internal purification campaigns. Among the metabolites with increased production, we could identify the presence of curvicollide A/B (*m*/*z* 432; C_26_H_40_O_5_) and fusidic acid (*m*/*z* 516; C_31_H_48_O_6_), and more tentativelly (matching of their molecular formulas with the commercial database Dictionary of Natural Products, DNP): pyrophen (*m*/*z* 309; C_18_H_17_NO_5_), cyclo-isoleucyl-leucyl-isoleucyl-leucyl (*m*/*z* 452; C_24_H_44_N_4_O_4_), melledonal C (*m*/*z* 497; C_24_H_29_ClO_8_) and rhizoxin S (*m*/*z* 613; C_34_H_47_NO_9_), previously described to be produced by a endosymbiotic bacteria in *Rhizopus* spp. On the contrary, when compared to the control condition, the production of several secondary metabolites was repressed in the presence of hydralazine (**2**). These melecules were identified tentatively, by comparison to our databases and DNP, as monascuspyrone (*m*/*z* 340; C_19_H_32_O_5_), pleurotin (*m*/*z* 354; C_21_H_22_O_5_), roseotoxin B (*m*/*z* 591; C_30_H_49_N_5_O_7_) and 12-hydroxy-8,10-octadecadienoic acid (*m*/*z* 653; C_18_H_32_O_3_). It is important to mention that all these molecules were only identified tentatively and future chemically directed purifications and HRMS/NMR confirmations are needed for a definitive confirmation.

Most differential ions detected with the volcano-plots methodology were not found in our chemical de-replication databases and could not be identified. These ions, according to their highest to lowest statistical significance *p*-value, included, for the production method A) (− +): *m*/*z* 243–244, 185, 374–375, (+ +): *m*/*z* 266–267–268, 243–244–245, 185–186–187; Production method B) (− − +): *m*/*z* 185–186, 157, 243, 247 and 225, (− + +): *m*/*z* 185–186, 171, 243, 247 and 227; and production method C) (− − +): *m*/*z* 185–186, 243 and 431, (− + +): 185–186, 243, 225, 171 (continuous ions, that are listed here together with hyphens, may belong to high intensity low resolution LC/MS signals detected in the raw data as contiguous *m*/*z* ions). In general, some of these most outlier ion masses were induced independently of the production method applied, being four of them the most statistically significative: *m*/*z* 171, 185–186, 225–226–227 and 243–244–245. These four sets of ions were studied then by UHPLC/HRMS-MS and their molecular formulae identified as C_9_H_6_N_4_ (**9**), C_10_H_8_N_4_ (**10**), C_13_H_14_N_4_ (**11**) and C_14_H_16_N_2_O_2_ (**12**). Interestingly the first three outlier molecules presented a molecular formula closely related to that of hydralazine (C_8_H_8_N_4_, **2**), and could not be found also in public nor commercial natural products databases. Further scale up and purification studies were setup for their identification.

**Figure 6:**
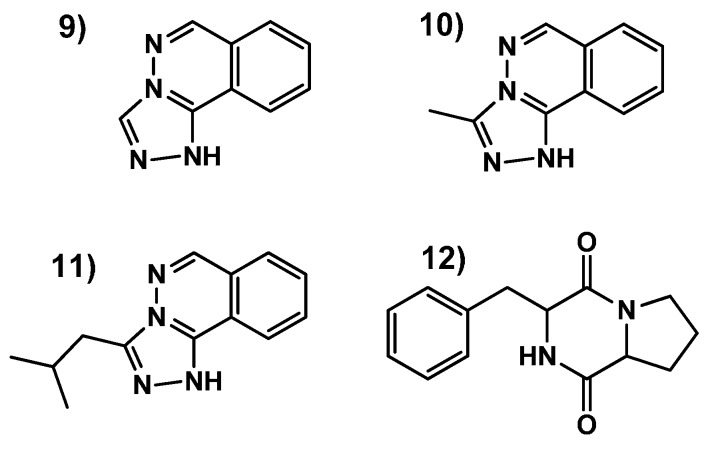
Most differential metabolites detected by the Volcano-plots methodology described, from the biotransformation (**9**–**11**), or the elicitor induction (**12**), of the epigenetic modifier hydralazine (**2**) on the fermentation broth of the endophyte fungus CF-285353.

Medium volume (600 mL) fermentations of the strain in the presence and absence of hydralazine (**2**) were extracted and fractionated for the isolation and identification of these differential molecules. HRMS/NMR results indicated that *m*/*z* 171, *m*/*z* 185–186, and *m*/*z* 227 ions corresponded to biotransformation products (**9**–**11**) of hydralazine (**2**) ocurring in the fermentation broth. On the other hand, the molecular formula C_14_H_16_N_2_O_2_, deduced from the ions *m*/*z* 243–244–245, that could be associated to 19 possible molecules in the natural products databases, was finally purified and identified as the diketopiperazine natural product cyclo(phenylalanyl-prolyl) (**12**) by HRMS and NMR data (Figure 6 and Supplementary Materials).

#### 2.3.4. *Dothiora* sp. Biomarkers Related to Growth Morphology

The differential growth morphologies observed in the strain CF-285353 and the availability of the metabolomic tools implemented for the evaluation of the epigenetic modifiers effects, also prompted us to perform the comparison of the profiles generated by the strain in the different morphologies, with the aim of identifing possible morphology biomarkers that could help us to monitor and fine tune future *Dothiora* sp. fermentations.

**Figure 7:**
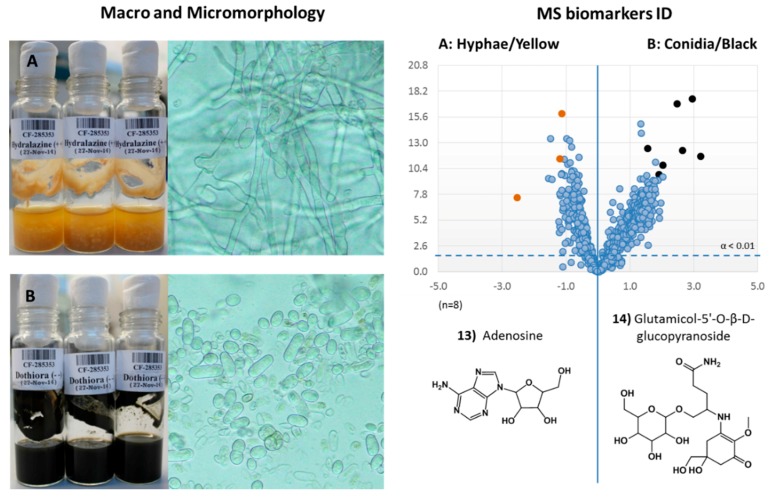
Volcano-plots representation for hyphae/yellow (**A**) *vs.* conidia/black (**B**) *Dothiora* sp. growth morphologies (−log 10 of *t*-test statistical *p*-value in *y*-axis *vs.* −log 2 of ion masses areas ratio in *x*-axes; *n* = 8), and the most differential SMs produced for each morphology determined by UHPLC-HRMS.

A representative population of eight CF-285353 extracts that presented the yellow hyphal morphology were compared with a similar population of extracts that presented the black conidial morphology, by using the volcano-plots approach (Figure 7). Statistically, and with a 99% of confidence, 713 mass ions were present in the conidial growths with significative higher titers than in the corresponding hyphal growths. On the other hand, 237 mass ions were present significatively in higher amounts in the hypha than in the conidia growth. Additionally, clear outlier populations of ions were observed for each condition, highlighting several SM pathways differentially expressed according to the fermentation morphology presented by the fungus.

The outlier ion masses with more significative abundance in the hyphal morphology were *m*/*z* 268, 269 and 226, whereas significative abundant ion masses in the conidial morphology were *m*/*z* 222, 465, 486 and 503. Both sets of components were studied, identified and their structures confirmed by UHPLC/HRMS-MS to correspond respectively to the primary metabolite adenosine (**13**) and the natural product mycosporin: glutamicol-5′-*O*-β-d-glucopyranoside (**14**) (Figure 7A,B).

Previous studies on the morphology, growth kinetics, and main chemical components of solid or liquid cultures of *Tolypocladium* fungi isolated from wild *Cordyceps sinensis*, showed that the *in vitro* hyphae mycelium of *Tolypocladium* presented much higher contents of adenosine (1116.8 μg *vs.* 264.6 μg) than when the fungus was directly isolated from *C. sinensis* [18]. Similarly to what was observed for *Tolypocladium*, adenosine could represent a potential biomarker of the hyphae growth morphology state as observed in our strain. Previous work by Pirttila *et al.* [19] suggests that the adenosine secretion of endophytes microorganisms may play an important role in the morphological development of the host plant and therefore, it could be playing a role in the secondary metabolism of the endophytes microorganism.

In the case of mycosporins, extremophilic microcolonial fungi have been described to constitutively synthesize considerable amounts of mycosporins, also known to be involved in morphogenesis and sporulation [20,21,22]. Many reports regarding different yeast species indicate the ability of these fungi to synthesize mycosporin-like amino acids, which have been also proposed to reflect phylogenetic relationships among species, suggesting their utility in yeast systematics. In addition, mycosporins have also been described in the extracellular matrix and in the outer cell wall layers of microcolonial fungi, in which they mediate a wide range of intracellular reactions. They are present in the mucilage that surrounds conidia of some fungi, confirming its direct presence in cultures with this morphology, with no evidences of their presence inside the conidia [23]. In some fungi, mycosporins are also described to protect conidia from solar radiation during atmospheric dispersal and prevent untimely germination [24] prolonging their survival. Therefore, the modulated production of these molecules by yeasts represents an interesting subject for further research due to its ecological, taxonomical and biotechnological implications [25]. For example, cosmetics applications as UV protectors and activators of cell proliferation, with potential therapeutic properties, could be interesting for other commercial developments [26].

Both detected molecules, adenosine (**13**) and mycosporin glutamicol-5′-*O*-β-d-glucopyranoside (**14**), confirmed in the literature as related to these morphology growth stages of some fungi, also corroborate the volcano-plots methology as a suitable approach for a succesful identification of metabolites produced differentially between two fermentation conditions, allowing a fast and robust approach for the identification of fermentation biomarkers.

## 3. Experimental Section

### 3.1. Isolation Cultures and Characterization

Standard indirect isolation techniques were followed to isolate the endophytes. Stems or leaves removed from each plant were cut into pieces of approximately 5 mm. These pieces were surface-disinfected sequentially through washing with 95% ethanol (30 s), 25% household bleach (1 min) and 95% ethanol (30 s). 10 pieces of each vegetal sample were aseptically transferred to a Petri dish with CMA supplemented with streptomycin sulfate and oxytetracycline (50 mg/mL) [14]. Epiphyte fungi were also directly isolated from cleistothecia or conidiophores formed on plants by incubation in moist chambers. Isolates were cultured in YM agar (malt extract 10 g, yeast extract 2 g, agar 20 g, 1000 mL distilled H_2_O), to study their macro- and microscopic characteristics. Strains designated with an ID (e.g., CF-282341) were preserved as frozen conidia and mycelia in 10% glycerol at −80 °C and are maintained in the culture collection of Fundación MEDINA (www.medinadiscovery.com). DNA extraction, PCR amplification and DNA sequencing were performed as previously described by Bills *et al.* in 2012 [14].

### 3.2. Epigenetic Modifiers Stock Solutions

Seven epigenetic modifiers were selected for its addition into inocula and fungal fermentations (Figure 1): two potent inhibitors of DNA methylation [(5-azacytidine (A2385, Sigma-Aldrich, St. Louis, MO, USA) and hydralazine hydrochloride (H1753, Sigma-Aldrich)], an inhibitor of sirtuins [nicotinamide (72340, Sigma-Aldrich)], a sirtuins activator [quercetin (Q4951, Sigma-Aldrich)] and three histone deacetylase (HDAC) inhibitors [suberohydroxamic acid (SBHA) (390585, Sigma-Aldrich), sodium butyrate (303410, Sigma-Aldrich) and valproic acid (P4543, Sigma-Aldrich)].

### 3.3. Inocula Preparation

The seed medium for all inocula was SMYA (DIFCO neopeptone 10 g, maltose 40 g, DIFCO yeast extract 10 g, agar 4 g, distilled H_2_O 1000 mL). For the control inocula five mycelial discs of fungal strains grown on YM agar at 22 °C for 7 days were cut from each 60 mm plate with a sterile “Transfer Tube” (Spectrum Laboratories, Rancho Dominguez, CA, USA). Mycelia discs were extruded from the “Transfer Tube” and crushed in the bottom of inocula tubes (25 × 150 mm) containing 12 mL of SMYA and two cover glasses (22 × 22 mm). Tubes were incubated on an orbital shaker (200 rpm; 1.5 cm throw), where rotation of the cover glasses continually sheared hyphae and mycelial disc fragments to produce nearly homogenous hyphal suspensions consisting of minute hyphal aggregates and fine mycelia pellets [27].

For each epigenetic modifier treatment during inocula, SMYA medium was prepared and each epigenetic modifier dissolved in DMSO or distilled water was aseptically added to each tube to attain for a final concentration of 100 μM. Tubes were then incubated for 7 days at 22 °C (Figure 3A). When pre-inocula was prepared from agar plugs (Figure 3B), the incubation time was also 7 days. When cryo-tubes were prepared, aliquots of 0.8 mL from this biomass were added to cryo-tubes containing 0.8 mL of glycerol and frozen at −80 °C. Along three consecutive batches (weeks), a cryo-tube per week was then thawed and added to Petri dishes containing YME agar medium to be incubated for 7 days. Agar plugs mycelia were then used to generate a new inocula for to be fermented for 7 days in SMYA before inoculating the 40 mL scintillation vials containing the production medium with or without the epigenetic modifier (Figure 3C).

### 3.4. Production Fermentations

The base production medium for all fermentation conditions was YES (DIFCO yeast extract 20 g, sucrose 150 g, MgSO_4_·7H_2_O 0.5 g, trace elements 1 mL (ZnSO_4_·7H_2_O 1 g/100 mL and CuSO_4_·5H_2_O 0.5 g/100 mL) and distilled H_2_O 1000 mL), a rich and clear medium that allows easy identification of differences in color, morphology and biomass [28]. The control condition was YES dispensed at 10 mL in 40 mL EPA vials, inoculated with 0.3 mL of mycelia inoculum, and shaked at 220 rpm [14]. Epigenetic modifiers dissolved in DMSO or distilled water were aseptically added to each vial to attain a final concentration of 100 μM [15]. All fermentations were then incubated for 7–14 days at 22 °C to equivalent biomass generation (Figure 3A–C).

### 3.5. Chemical Extraction

After harvesting, 9 mL whole broths were extracted by adding 9 mL of acetone using a Multiprobe II robotic liquid handler (PerkinElmer, Waltham, MA, USA) and shaking at 220 rpm for 1 h. After centrifugation, 12 mL of supernatant from each vial were transferred to glass tubes containing 0.6 mL of DMSO and mixed. Solvent was evaporated under a heated nitrogen stream to a final volume of 3 mL (80/20 water/DMSO solution) and a final concentration of 2 × WBE (whole broth equivalents). Each fermentation batch included extracts from control culture media to discriminate their components [28].

### 3.6. UHPLC-UV Profile Analysis

Chemical profiles of fermentation extracts (4 μL) were analyzed using an Agilent 1290 Infinity UHPLC-UV (Santa Clara, CA, USA). A Kinetex C-18 (1.7 μm, 2.1 × 150 mm) column from Phenomenex (Torrance, CA, USA), a 10 min gradient from 1% to 99% (*v/v*) of acetonitrile in water, with 1.3 mM ammonium formate and 1.3 mM trifluoroacetic acid as chromatographic modifiers, a flow rate of 315 μL/min, a controlled temperature of 40 °C, and UV detection at 210, 280 and 340 nm were used for each analysis. An inert internal control was present in each sample to individually validate and normalize if necessary retention times of each chromatographic run. Additional methanol blanks were injected every ten samples for joint monitoring of each analytical batch [28].

### 3.7. LC/MS Data and Metabolomics Evaluation

Samples selected (2 μL) were analyzed by HPLC-MS. LC analysis was performed on an Agilent 1200, using a Zorbax SB-C8 column (2.1 × 30 mm), maintained at 40 °C and with a flow rate of 300 μL/min. Solvent A consisted of 10% acetronitrile and 90% water with 0.01% trifluoroacetic acid and 1.3 mM ammonium formate, while solvent B was 90% acetronitrile and 10% water with 0.01% trifluoroacetic acid and 1.3 mM ammonium formate. The gradient started at 10% B and went to 100% B in 6 minutes, kept at 100% B for 2 min and returned to 10% B for 2 min to initialize the system. Full diode array UV scans from 100 to 900 nm were collected in 4 nm steps at 0.25 sec/scan. Mass spectrometry acquisition was performed on an Agilent MSD 1100 mass spectrometer for generating the metabolomics raw data.

Volcano-plots were calculated according to Hur *et al.* [13] but using each low resolution *m*/*z* as a component, and without introducing at this step a deconvolution for simplifying all *m*/*z* observed for a single metabolite into a single component. This simplified strongly the IT investment required for data management and processing, highlighting equally the differential components observed for each treatment comparison. UHPLC/HRMS identification and characterization of the components of interest included all those features in a following step, once the ion masses of interest were identified.

In the *x*-axis, volcano-plots depict the −log 10 of the *t*-test statistical *p*-value from comparing two populations of the same component (*m*/*z* in our study) treated with the two conditions of interest, whereas in the *y*-axis, a −log 2 representation on the ratios of the areas of one condition among the other is plotted for the same component [29]. In these volcano-plots, scatter plots (*m*/*z*) 1.3 units above in the *y*-axis presented significant differences among both conditions with a 95% of statistical confidence (α = 0.05). Plots above 2 units presented a 99% (α = 0.01) of significant differences between both treatments. For an easy visual interpretation, 1.3 was used as the main cross mark in the scatter-plots depicted in our study. The number of mass ions statistically different by higher production for each growth condition is indicated for 95%: 99% of statiscical confidences (α < 0.05 and α < 0.01 respectively). On the other hand, units in the *y*-axes are correlated to relative sequential abundances of 200%–400%–800% of one condition compared to the other. The ratio also helps in minimizing/normalizing the diferential ionization signals than can be observed for each metabolite [13].

### 3.8. UHPLC-HRMS Database Matching of Known Metabolites and Antibiotics

A Bruker maXis HR-QTOF mass spectrometer (Bruker Daltonics GmbH, Bremen, Germany) coupled to the previously described UHPLC system was used for characterization and identification of specific metabolites. Ionization of the eluting solvent were obtained using the standard ESI sources adjusted to a drying gas flow of 11 L/min at 200 °C and a nebulizer pressure of 40 psig. The capillary voltage was set to 4000 V. Mass spectra were collected from 150 *m*/*z* to 2000 *m*/*z* in positive mode. Database matching was performed by comparing retention time and exact mass generated with the Bruker maXis, of extracted components from the samples under study, with the retention times and exact masses from known metabolites stored in a database acquired under the same exact LC-HRMS conditions.

## 4. Conclusions 

The systematic addition of small-molecule epigenetic elicitors during the inocula stages in several fungi of the study, determined the activation of biosynthetic pathways otherwise silent when grown under standard conditions. The increase in production titers of global SM profiles that was observed in some of the strains was slightly higher when the elicitors were present since the inoculum stage. A UHPLC-UV screening of the use of epigenetic elicitors in multiple conditions highlighted the most interesting combinations of strain/elicitor/fermentation methodology. The Volcano-plots methodology proved to be a robust way to identify and quantify in detail these effects in fungal microbial fermentations.

Among the seven epigenetic modifiers studied, hydralazine resulted the most effective one for a differential generation of SMs profiles in the CF-285353 *Dothiora* sp. strain. An extensive study on the differential profiles generated for this strain by hydralazine indicated 4 molecules as the most significative ones in differential production. Scale-up fermentations of CF-285353 identified three of these molecules as biotransformation products of hydralazine, and determined cyclo(phenylalanyl-prolyl) as a natural product whose production was clearly induced by the addition of the epigenetic modifier. Differences on the growth morphology presented by the fungus CF-285353 were also identified and quantified by the metabolomic approach described, allowing the identification of adenosine and the mycosporin glutamicol-5′-*O*-β-d-glucopyranoside as conidia/hyphae morphology biomarkers respectively. Evaluation throughout metabolomics on the other two strains highlighted in the modifiers screening (CF-277101 and CF-282001) is currently underway for a better understanding of the effect of epigenetic modifiers in the fermentation of this population of fungal endophytes.

Results also indicated the possibility that, in some cases, the epigenetic modifier added may not only be inducing the expresion or activation of criptic pathways, but can also suffer biotransformations. In both situations, the fermentation methodologies described, the systematic screening of epigenetic elicitors presented, the Volcano-plots metabolomics methodology applied for large sets of MS data, and the subsequent dereplication and identification of the differential components by HRMS, can result in a robust aproach for highlighting and quantifying both biotransformations and possible silent patways induced by small mollecule elicitors in fungal endophytes.

## Figures and Tables

**Table 1:** Host plant and geographical origin of the fungal strains isolated.

Family	Strain Code	Species	Host Plant	Origin	Genbank
*Dothideaceae*	CF-277039	*Kabatiella bupleuri*	*Bupleurum gibraltarium*	Presa de Quentar, (Granada)	JN886788
*Dothideaceae*	CF-277101	*Selenophoma* sp.	*Spartium junceum*	Fuente del Hervidero, (Granada)	JN886791
*Dothideaceae*	CF-280549	*Kabatiella* sp.	*Bupleurum spinosum*	Camino de los neveros (Granada)	KU295574
*Dothideaceae*	CF-285353	*Dothiora* sp.	*Launaea arborescens*	Tabernas (Almeria)	KU295575
*Dothideaceae*	CF-285359	*Kabatiella* sp.	*Asparagus horridus*	Tabernas (Almeria)	KU295576
*Dothideaceae*	CF-285463	*Selenophoma juncea*	*Salsola oppositifolia*	Tabernas (Almeria)	KU295578
*Dothideaceae*	CF-285762	*Aureobasidium pullulans*	*Inula chrihtmoides*	Punta Entinas (Almeria)	KU295579
*Chaetothyriaceae*	CF-285360	*Knufia* sp.	*Asparagus horridus*	Tabernas (Almeria)	KU295577
*Phaeosphaeriaceae*	CF-285372	*Chaetosphaeronema* sp.	*Anabasis articulata*	Tabernas (Almeria)	KU295581
*Planistromellaceae*	CF-282001	*Loratospora* sp.	*Retama sphaerocarpa*	Albuñuelas (Granada)	KU295580
*Sporormiaceae*	CF-282341	*Preussia* sp.	*Dittrichia viscosa*	Alhendin (Granada)	KU295582
*Sporormiaceae*	CF-285375	*Preussia australis*	*Launaea arborescens*	Tabernas (Almeria)	KU295583
*Xylariaceae*	CF-285461	*Xylaria* sp.	*Thymelaea hirsuta*	Tabernas (Almeria)	KU295584

**Table 2:** Effect of the different epigenetic treatments on the UHPLC-UV 210 nm chemical profiles of the fungal endophytes of the study.

Strain ID	5-Azacitidine (1)		Hydralazine (2)		Nicotinamide (3)		Quercetin (4)		SBHA (5)		Sodium Butyrate (6)		Valproic (7)	
	(− +)	(+ +)	(− +)	(+ +)	(− +)	(+ +)	(− +)	(+ +)	(− +)	(+ +)	(− +)	(+ +)	(− +)	(+ +)
CF-277039	-	-	-	-	-	-	-	-	*p*	*m*	*pp*	*mp*	*m*	m
**CF-277101**	***p***	***p***	***p***	***p***	***p***	***p***	***p***	***p***	***p***	***dmp***	***p***	***p***	***p***	**p**
CF-280549	*m*	*m*	*m*	*-*	*m*	*-*	*m*	*m*	*m*	*mp*	*m*	*-*	*p*	-
**CF-285353**	**-**	***ddmpp***	***-***	***ddmpp***	***-***	***-***	***-***	***ddmpp***	***p***	***m***	***p***	***-***	**-**	**m**
CF-285359	-	*m*	*-*	*m*	*-*	*m*	*m*	*-*	*-*	*m*	*-*	-	-	m
CF-285463	-	*mp*	*-*	*-*	*-*	*mp*	*-*	*mp*	*-*	*mp*	*-*	*mp*	-	mp
CF-285762	-	-	-	-	-	-	-	-	-	-	-	-	-	-
CF-285360	-	-	-	-	-	*p*	*-*	*-*	*-*	*p*	*-*	*p*	-	-
CF-285372	*p*	*p*	*p*	*p*	*-*	*-*	*-*	*p*	*p*	*p*	*p*	*p*	*-*	-
**CF-282001**	***pp***	***p***	***ppp***	***pp***	***-***	***-***	***p***	***dppp***	***p***	***pp***	***pd***	***ddpp***	***m***	**m**
CF-282341	-	-	-	-	-	-	-	-	-	-	-	-	-	-
CF-285375	*p*	*p*	*-*	*-*	*-*	*-*	*-*	*-*	*-*	*-*	*p*	-	-	-
CF-285461	-	-	-	-	-	-	-	-	-	-	-	-	-	-

***m****: changes in morphology; **p-pp-ppp**: increasing changes in production titers; **d-dd**: increasing changes in chemical diversity.*

## Supplementary Materials

Supplementary materials can be accessed at: http://www.mdpi.com/1420-3049/21/2/234/s1.
