# Supplementary material for: Multicomponent Analysis of the Differential Induction of Secondary Metabolite Profiles in Fungal Endophytes
Source: Molecules. 2016 Feb 18;21(2):234. doi: 10.3390/molecules21020234 (PMC6272891; doi:10.3390/molecules21020234)
Supplement: Supplementary file 1 [file molecules-21-00234-s001.pdf]

Supplementary Materials: Multicomponent Analysis of the Differential Induction of Secondary Metabolite Profiles in Fungal Endophytes

Víctor González-Menéndez, Mercedes Pérez-Bonilla, Ignacio Pérez-Victoria, Jesús Martín, Francisca Muñoz, Fernando Reyes, José R. Tormo and Olga Genilloud

Isolation and Identification of Metabolites

The mycelium and fermentation broth (600 mL) was extracted by adding acetone (600 mL) and shaking at 220 rpm for 2 h. After filtration, the acetone extract was concentrated under reduced pressure to a final volume of 600 mL (100% water). The aqueous residue was loaded onto a SP207ss resin column (65 g, 32 × 100 mm) and eluted with an acetone/H_2O stepped gradient (10/90 for 6 min, 20/80 for 6 min, 40/60 for 6 min, 60/40 for 6 min, 80/20 for 6 min and 100/0 for 12 min, 10 mL/min, 20 mL/fraction) to give nineteen fractions. Target metabolites were identified by LCMS in these fractions. An aliquot (300 µL) of fraction FS010 was subjected to semi-preparative reversed phase HPLC (Zorbax SB-C_{18} column, 9.4 × 250 mm, 5 µm, 3.6 mL/min, UV detection at 210 and 280 nm) eluting with CH_3CN: H_2O, linear gradient from 5 to 100% CH_3CN in 63 min to yield a mixture of compounds **9** and **12** (t_R 26 min) and compound **10** (t_R 30 min). Fractions FS011 to FS014 were pooled and subjected to preparative reversed-phase HPLC (Zorbax SB-C_{18} PrepHT, 21.2 × 250 mm, 7 µm, 16 mL/min, UV detection at 210 and 280 nm) using H_2O + 0.1% TFA (solvent A) and CH_3CN + 0.1% TFA (solvent B), isocratic conditions of 5% B for 5 min and then a linear gradient from 5% to 100% B in 43 min to yield a fraction containing compound **11** (t_R 28 min) and major impurities.

A molecular formula of $C_9H_6N_4$ was assigned to compound **9** after analysis of its ESI-TOF spectrum (*m*/*z* 171.0657 $[M + H]^+$, calc. for $C_9H_7N_4^+$, 171.0664). The presence of a singlet at 8.95 ppm in the ^{1}H-NMR spectra of compound **9** along with two doublets at 8.18 ppm (H-6) and 8.60 ppm (H-9) and two triplets each integrating for one proton at 7.97 ppm (H-7) and 8.08 ppm (H-8) were assigned to the aromatic protons of a phtalazine moiety. In addition, a singlet observed at 9.40 ppm was assigned to the H-13 proton. The MS/MS analysis of compound **9** showed a quasimolecular ion at 144.0551 corresponding to $C_8H_6N_3^+$, due to the loss of the carbon and one of the nitrogen atoms of the triazol moiety, and consistent with the proposed structure of [1,2,4]triazolo[3,4-a]phthalazine.

Compound **10** displayed a pseudomolecular ion at *m*/*z* 185.0819 (calc. for $[M + H]^+$ 185.0827) corresponding to a molecular formula of $C_{10}H_8N_4$. It displayed similar chemical shifts as compound **9** for the protons and carbons of the phthalazine moiety. The major difference between the ^{1}H-NMR spectra of both compounds was the absence of the proton at 9.40 ppm in the spectrum of **9** and the presence of a methyl group at 2.81 ppm in the spectrum of **10**. The chemical shift of the methyl group in the carbon spectrum (9.7 ppm) indicated its linkage to a carbon that was identified as C-13. A cross peak between the methyl group and a carbon at 150.12 ppm observed in the HMBC spectrum supported this evidence. According to the MS/MS results, compound **10** displayed the same ion at 144.0559 as compound **9**, suggesting the same fragmentation pattern that is consistent with a structure of 3-methyl-[1,2,4]triazolo[3,4-a]phthalazine for this compound.

Compound **11** was assigned a molecular formula of $C_{13}H_{14}N_4$ by ESI-TOF (*m*/*z* 227.1280, calc. for $[M + H]^+$ 227.1286). The NMR spectra of this compound were very similar to those of compounds **9** and **10**, with the major difference being in the absence of the methyl group present in **10** and the presence of a doublet at 3.13 ppm accounting for methylene protons, together a multiplet corresponding to a proton in the aliphatic region. Additionally, this proton was coupled in the COSY spectrum to two aliphatic methyl groups and confirmed the presence of an isobutyl moiety in the structure. Finally this compound also presented a quasimolecular ion at *m*/*z* 144.0555 corresponding to $C_8H_6N_3^+$ in a MS/MS measurement, confirming the same structural pattern as for compounds **9** and **10**, and the structural proposal.

Compounds **9** and **10** have been previously reported as metabolites obtained by transformation of hydralazine by rat liver microsomes [1] as well as main products formed in human saliva and under gastric conditions when this drug was administered to patients suffering from hypertension [2].

The identification of compound **12** was straightforward from the molecular formula and proton and 2D-NMR spectra. Compound **12** displayed a pseudomolecular ion at *m*/*z* 245.1282 (calc. for $[M + H]^+$ 245.1206) corresponding to a molecular formula of $C_{14}H_{16}N_2O_2$ suggesting a diketopiperazine nature for the compound. The presence of a proline moiety was inferred from signals attributed to the presence of three broad methylene multiplets in the proton spectrum (δ_H 3.31–3.35 and 3.51–3.57 (C-3), δ_H 1.60–1.69 and 1.88–1.93 (C-4) and δ_H 1.60–1.69 and 2.01–2.06 (C-5)) and by H-H correlations observed in the COSY experiment. Proton in alpha for proline amino acid presents a chemical shift of 2.60–2.63 ppm that is significantly different to that expected for an amino acid alpha proton.
This upfield shift could be due to a conformational arrangement of the aromatic ring that leaves this proton closer to its inner part. Analysis of the NMR spectra also indicated that phenylalanine was the second amino acid residue, showing signals of methylene benzylic protons at δ_H 2.99 and 3.19 respectively (C-10) attached to a monosubstituted aromatic ring (δ_H 7.18–7.31). A proton at 4.20 ppm is attributed to the alpha proton for phenylalanine residue.

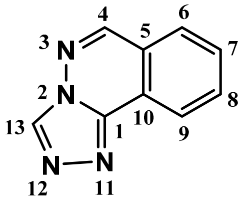

9

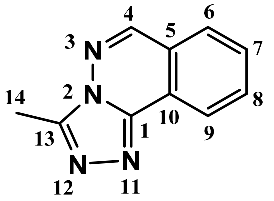

10

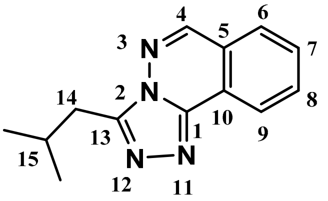

11

Table NMR. ^{1}H-NMR Data of Hydralazine and Compounds **9**, **10** and **11**.[a]

Position	Hydralazine	9	10	11
	δ_H, m (*J* in Hz)	δ_H, m (*J* in Hz)	δ_H, m (*J* in Hz)	δ_H, m (*J* in Hz)
1	--	--	--	--
2	--	--	--	--
3	--	--	--	--
4	8.78, s	8.95 s	8.93 s	8.95 s
5	--	--	--	--
6	8.16–8.18, m	8.18, dd (8.0, 1.1)	8.16, brd (8.0)	8.18, brd (7.5)
7	8.07–8.10, m	7.97, td (8.0, 1.1)	7.94, td (8.0, 1.2)	7.96, brt (7.5)
8	8.16–8.18, m	8.08, td (8.0, 1.1)	8.05, td (8.0, 1.1)	8.07, brt (7.5)
9	8.41, dd (8.2, 0.9)	8.60, td (8.0, 1.1)	8.54, dd (8.0, 0.7)	8.56, brd (7.5)
10	--	--	--	--
11	--	--	--	--
12	--	--	--	--
13	--	9.40, s	--	--
14	--	--	2.81, s	3.13, d (7.2)
15	--	--	--	2.32–2.36, m
16	--	--	--	1.04–1.06, m
17	--	--	--	1.04–1.06, m

[a] Measured in CD_3OD, Chemical shifts (δ) in ppm.

Table NMR. ^{13}C-NMR Data of Hydralazine and Compounds **9**, **10** and **11**.[b]

Position	Hydralazine [a]	9 [a]	10	11 [a]
	δC	δC	δC	δC
1	--	--	144.43	--
2	--	--	--	--
3	--	--	--	--
4	144.94	149.18	150.12	149.97
5	--	--	125.14	--
6	136.96	128.81	130.32	130.05
7	134.84	131.40	132.81	132.67
8	129.31	134.16	135.62	135.46
9	124.47	122.18	123.74	123.48
10	--	--	124.10	--
11	--	--	--	--
12	--	--	--	--
13	--	140.55	150.12	--
14	--	--	9.70	32.20
15	--	--	--	29.85
16	--	--	--	21.22
17	--	--	--	21.22

[a] Signal obtained from HSQC spectrum; [b] Measured in CD_3OD, Chemical shifts (δ) in ppm.

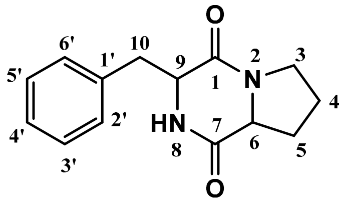

12

Table NMR. ^{1}H and ^{13}C-NMR [a] Data of Compounds **12**.[b]

Position	12	
	δH, m (J in Hz)	δC
1	--	--
2	--	--
3a	3.51–3.57, m	44.64
3b	3.31–3.35,[c] m	
4a	1.88–1.93, m	21.02
4b	1.60–1.69, m	
5a	2.01–2.06, m	28.33
5b	1.60–1.69, m	
6	2.60–2.63, m	57.66
7	--	--
8	--	--
9	4.20, t (4.7)	58.30
10a	3.19, dd (13.7, 4.7)	39.50
10b	2.99, dd (13.7,4.7)	
1′	--	--
2′	7.18–7.20, m	129.80
3′	7.30–7.31, m	128.48
4′	7.30–7.31, m	127.26
5′	7.30–7.31, m	127.76
6′	7.18–7.20, m	129.47

[a] Signal obtained from HSQC spectrum; [b] Measured in CD_3OD, Chemical shifts (δ) in ppm; [c] Obscured by solvent peak.

Reference

1. LaCagnin, L.B.; Colby, H.D.; O'Donnell, J.P. The oxidative metabolism of hydralazine by rat liver microsomes. *Drug Metab. Dispos.* **1986**, *14*, 549–554.
2. Noda, A.; Matsuyama, K.; Yen, S.-H.; Sogabe, K. Fate of Hydralazine in Man. I. Reactions under Gastric Conditions. *Chem. Pharm. Bull.* **1979**, *27*, 2820–2826.

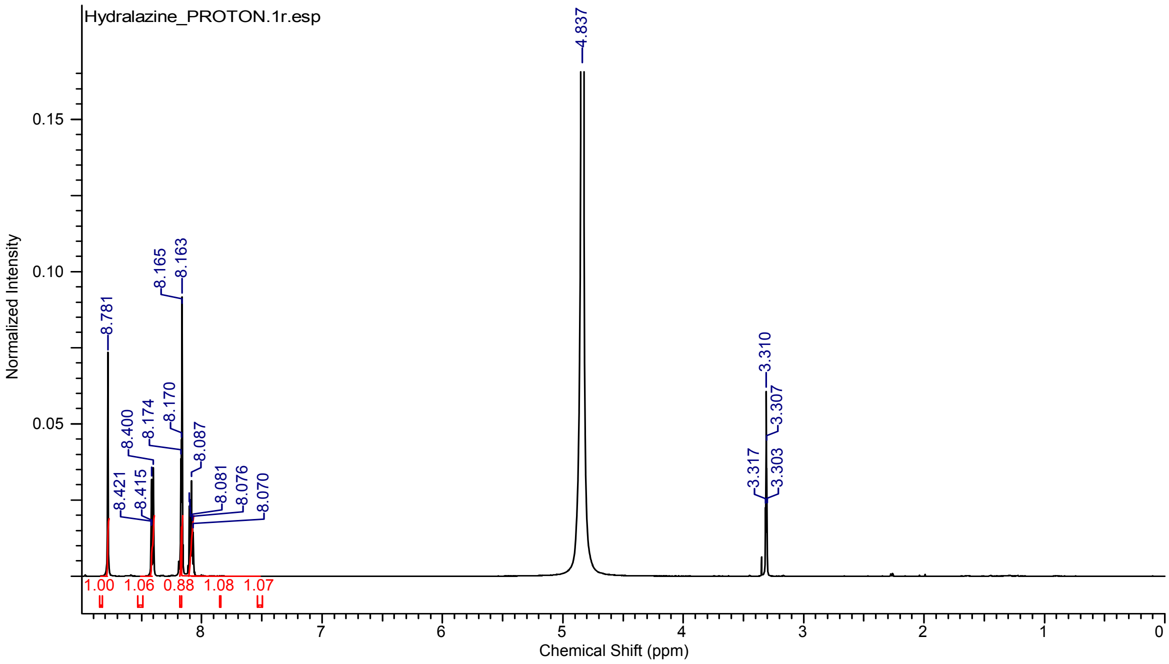

Figure S1. Hydralazine ^{1}H-RMN.

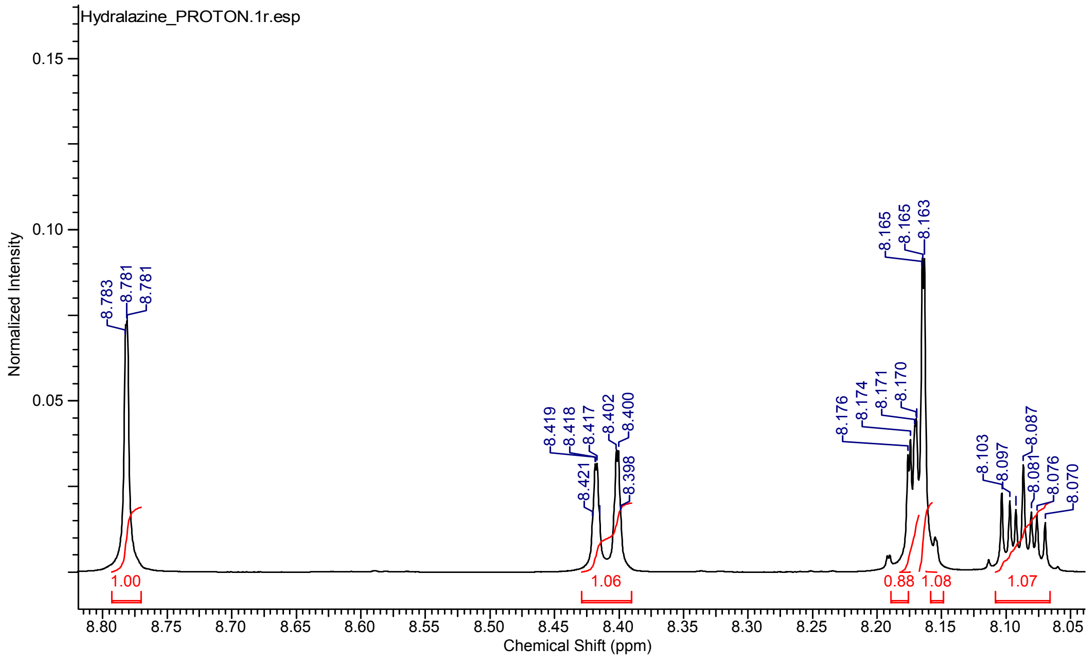

Figure S2. Hydralazine ^{1}H-RMN.

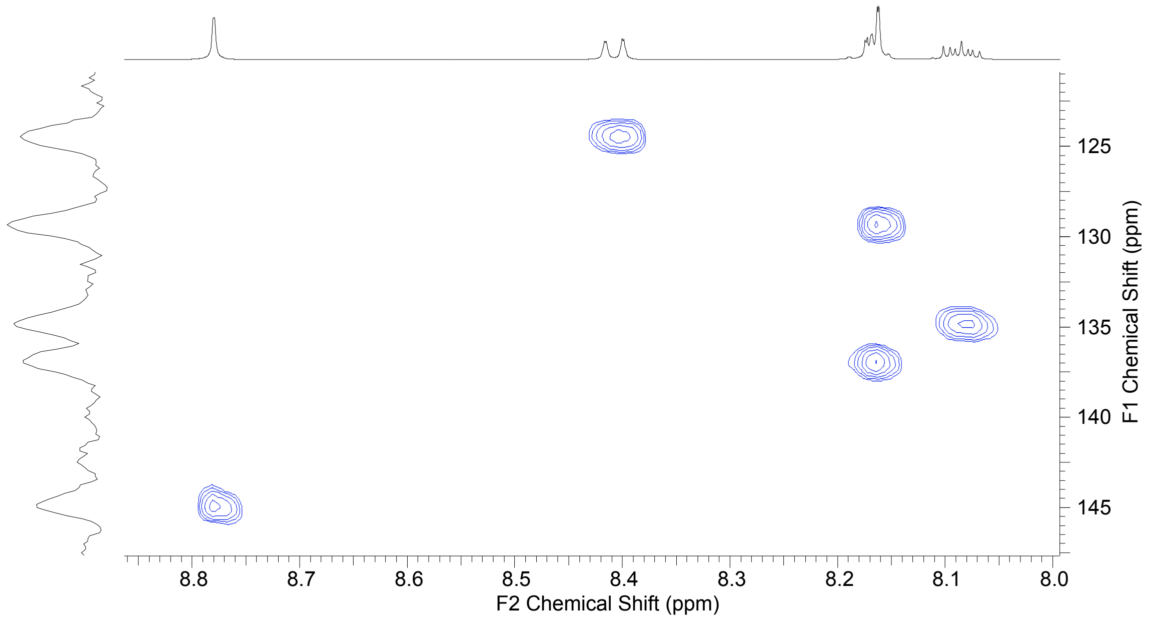

Figure S3. Hydralazine HSQC.

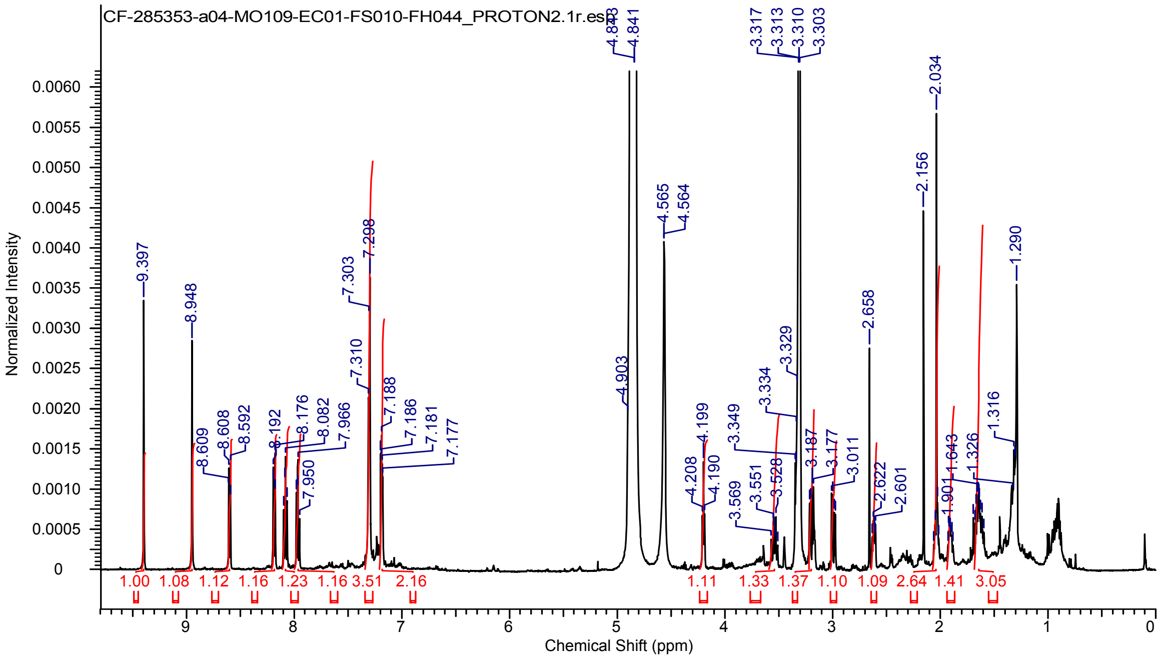

Figure S4. Compounds **9** and **12** ^{1}H-RMN.

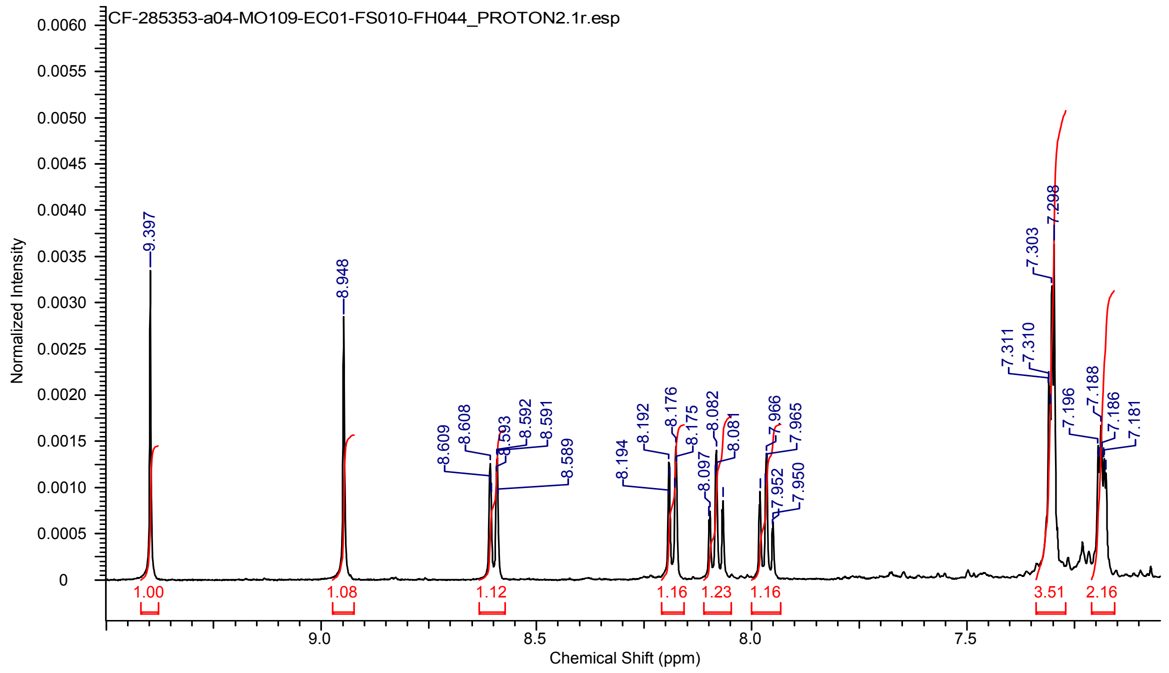

Figure S5. Compounds **9** and **12** ^{1}H-RMN.

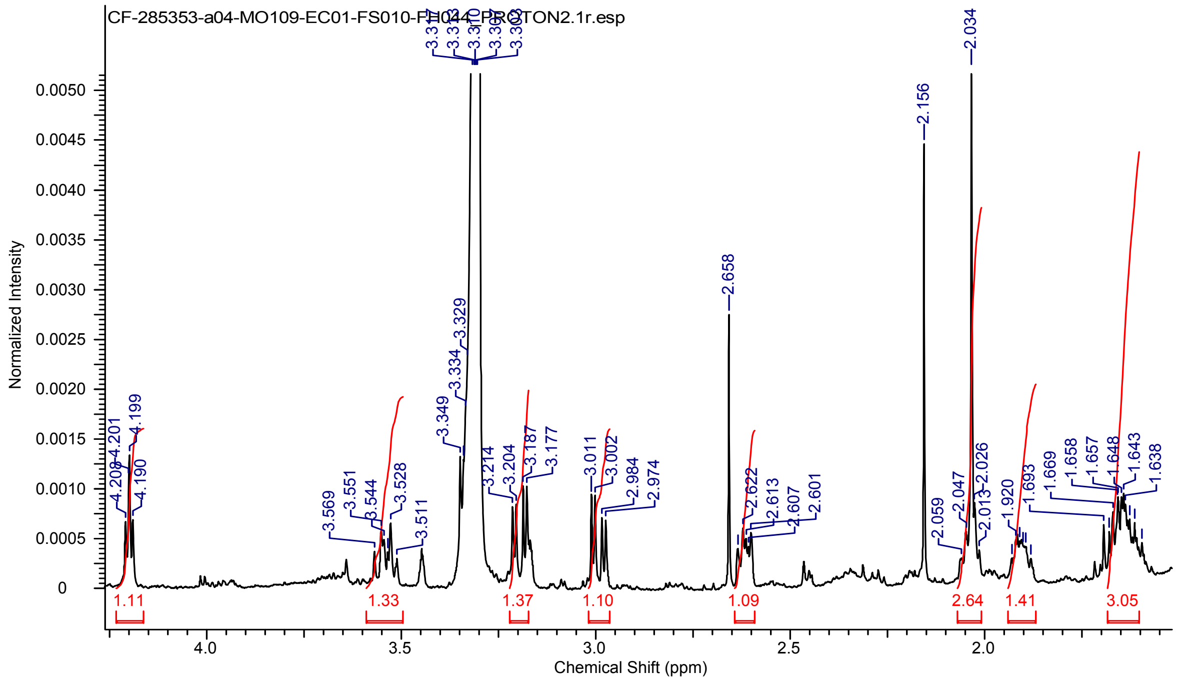

Figure S6. Compounds **9** and **12** ^{1}H-RMN.

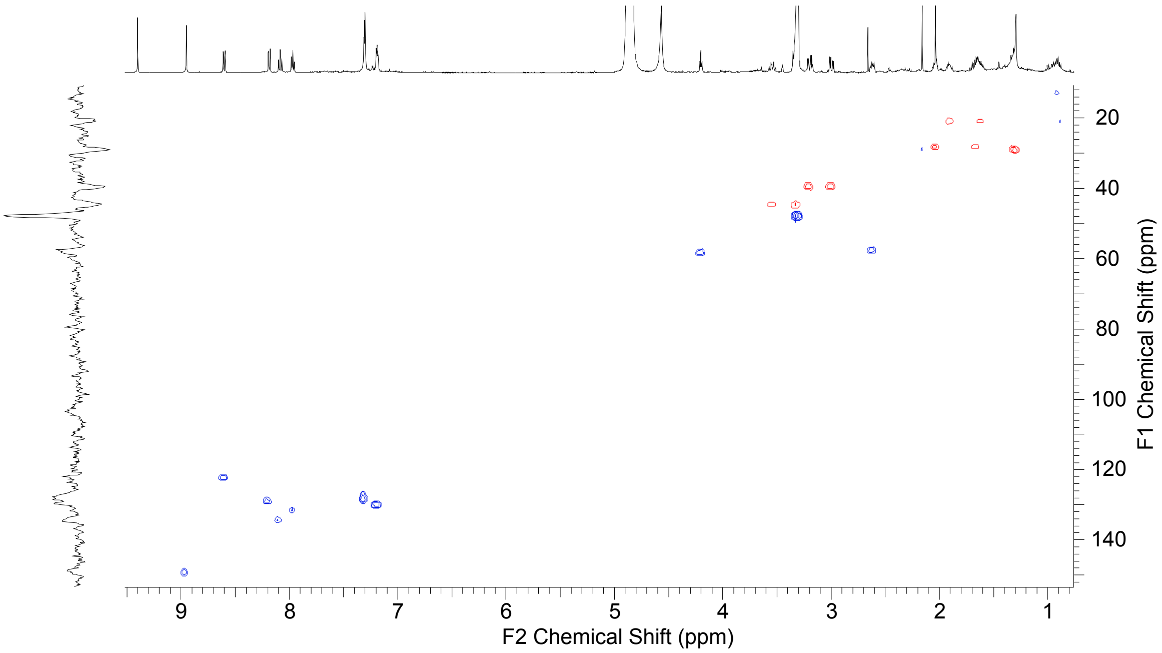

Figure S7. Compounds **9** and **12** HSQC.

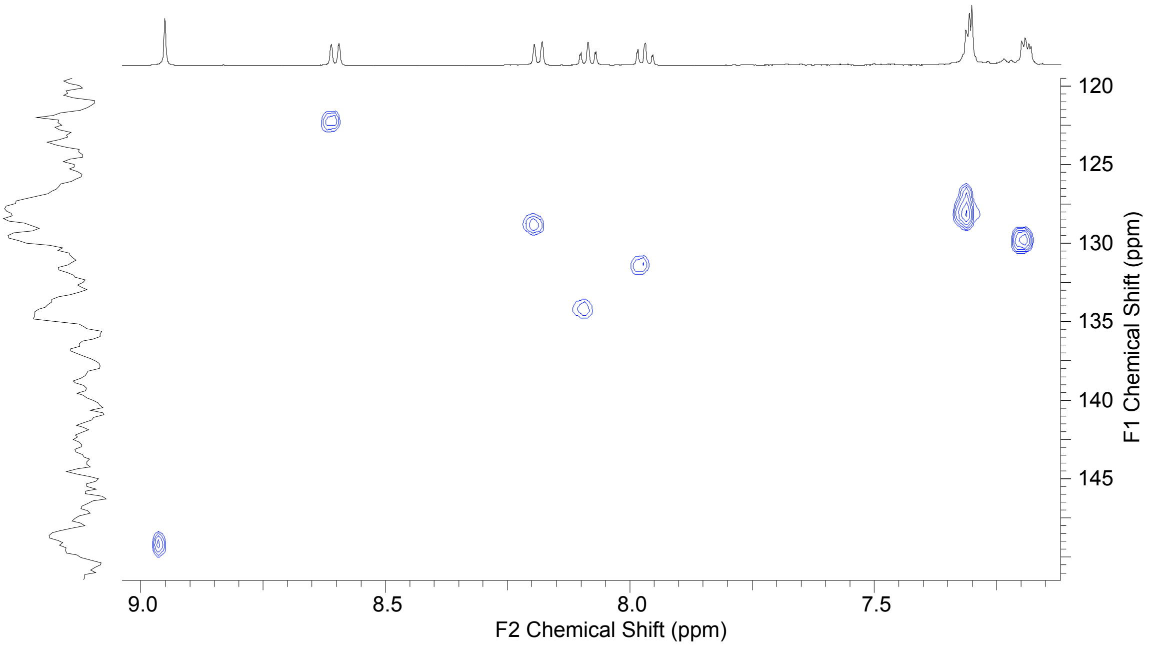

Figure S8. Compounds **9** and **12** HSQC.

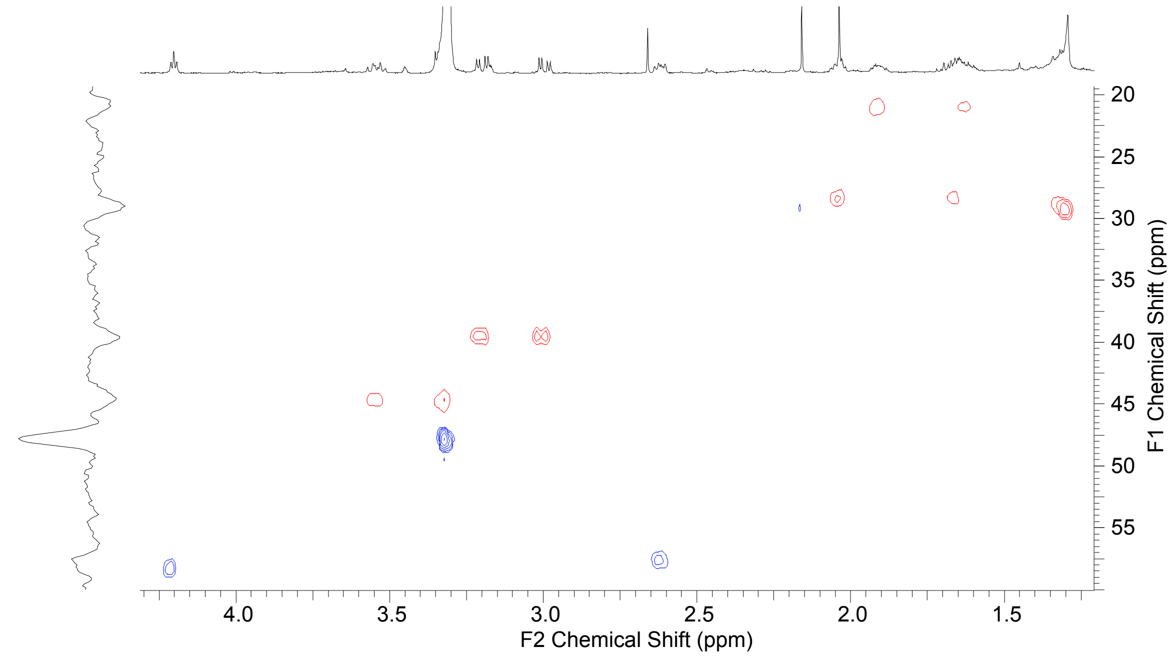

Figure S9. Compounds **9** and **12** HSQC.

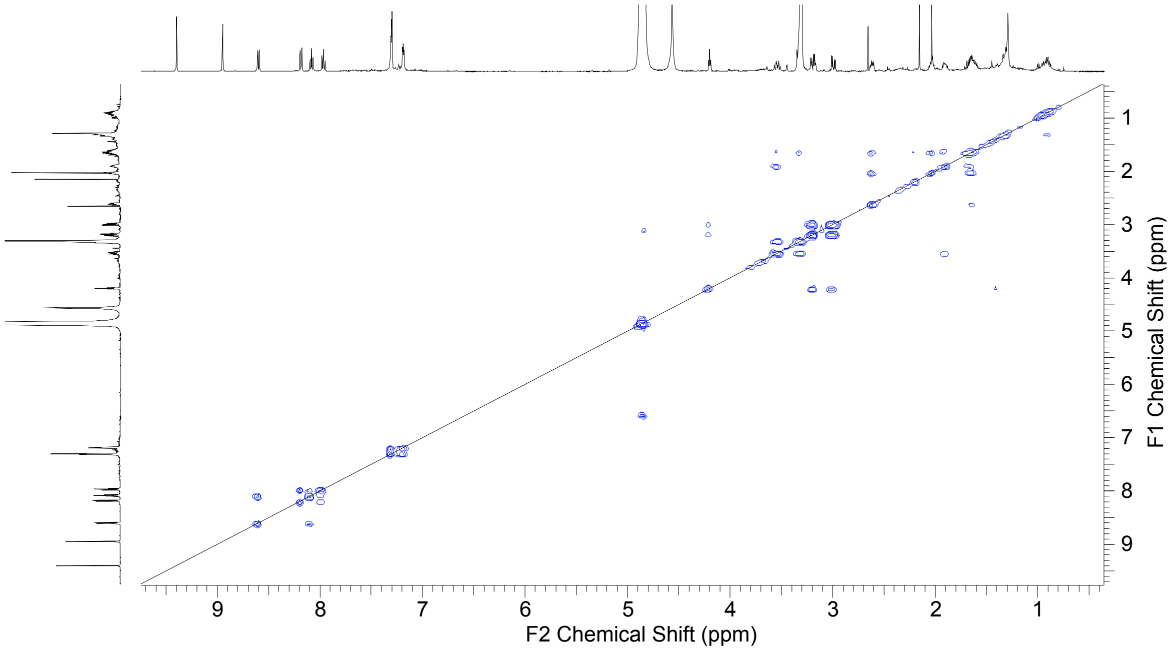

Figure S10. Compounds **9** and **12** COSY.

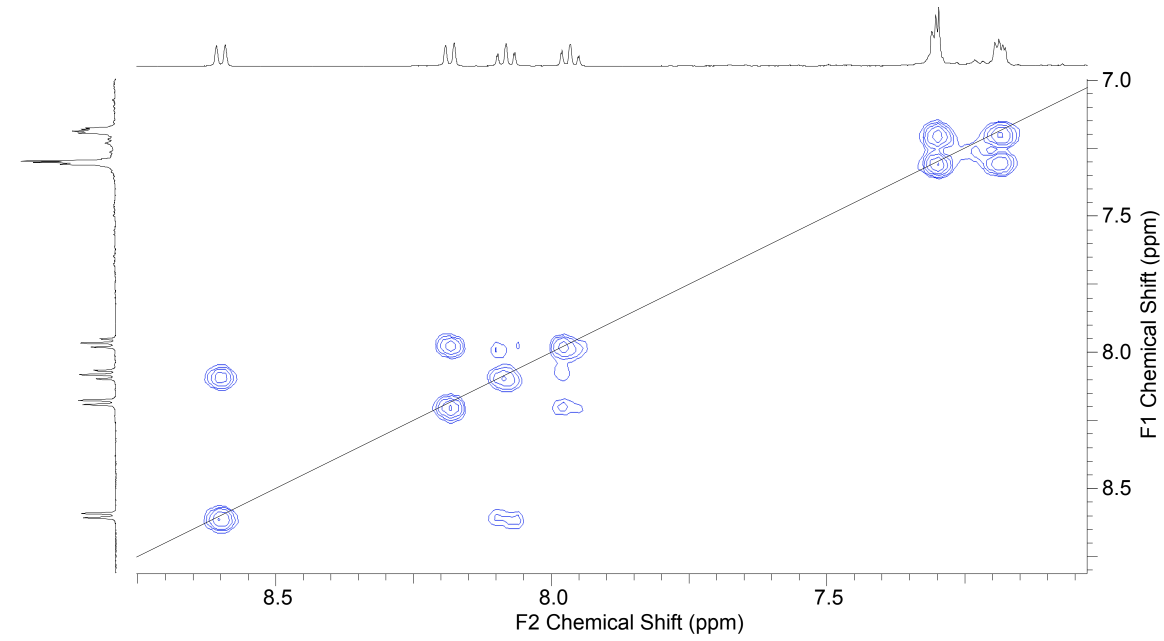

Figure S11. Compounds **9** and **12** COSY.

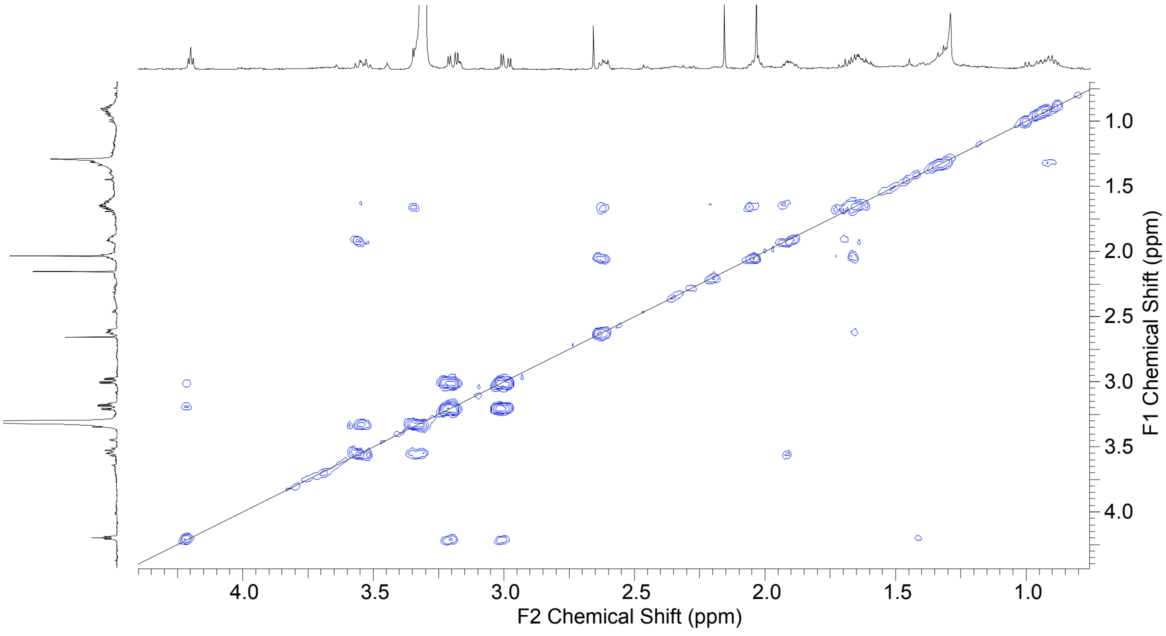

Figure S12. Compounds **9** and **12** COSY.

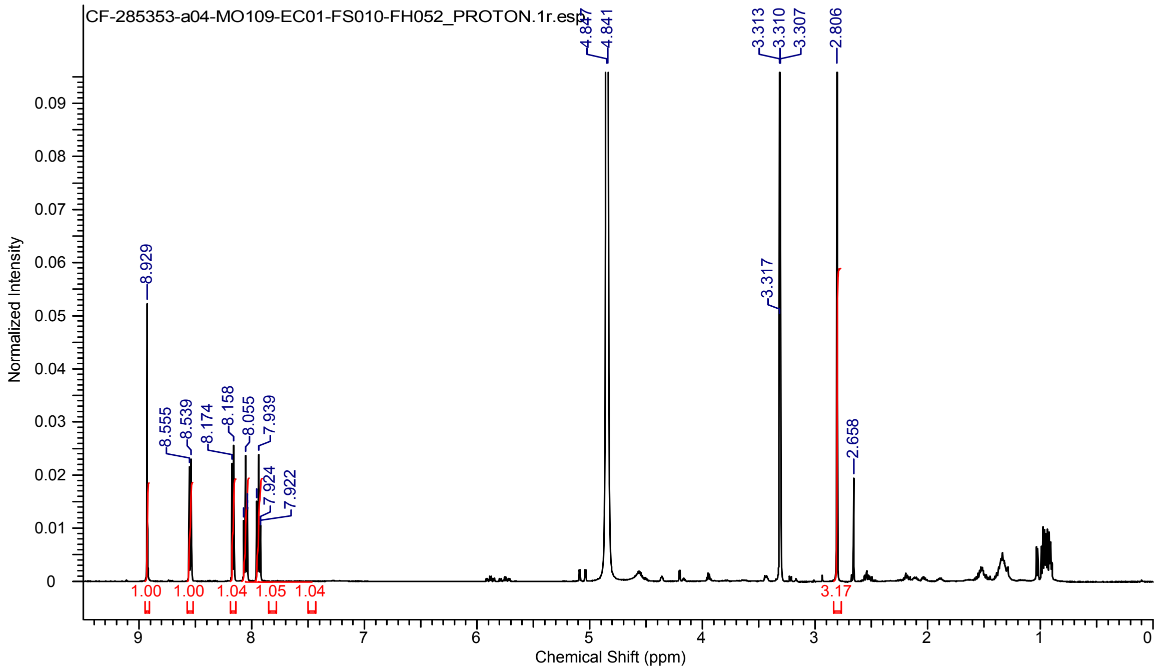

Figure S13. Compound **10** ^{1}H-RMN.

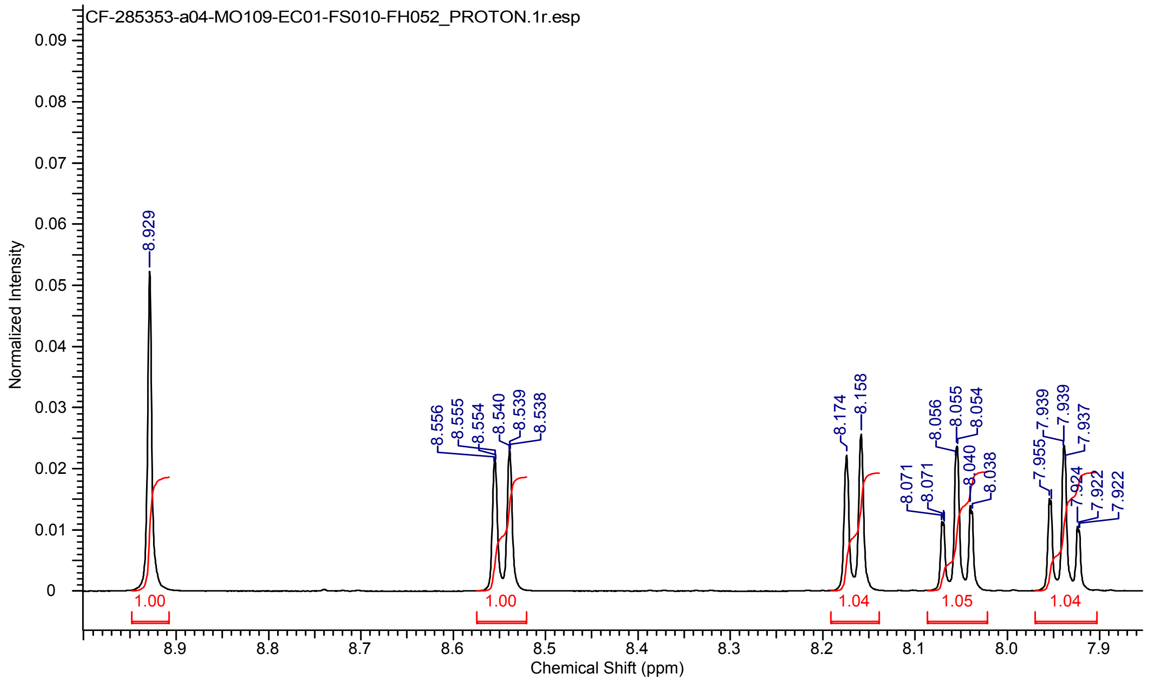

Figure S14. Compound **10** ^{1}H-RMN.

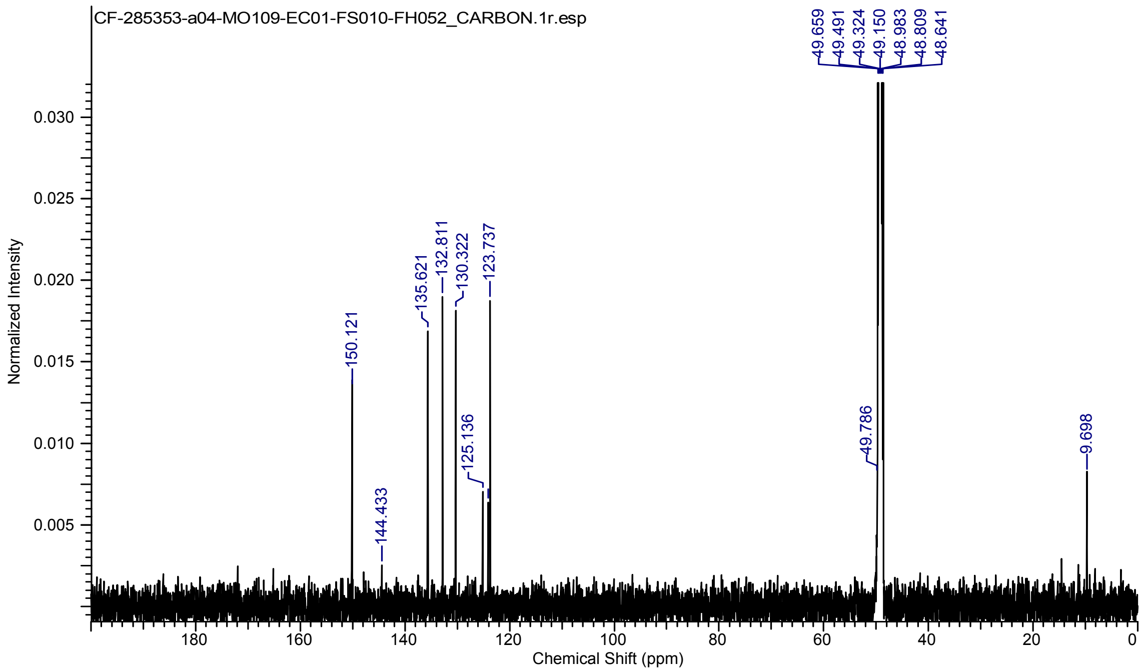

Figure S15. Compound **10** ^{13}C-RMN.

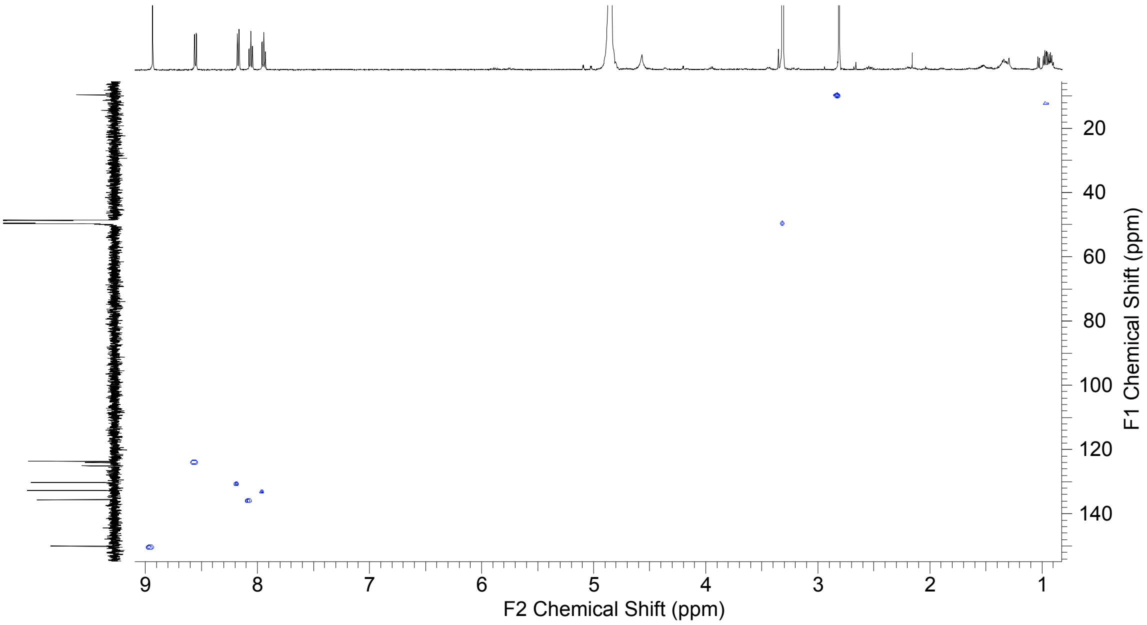

Figure S16. Compound **10** HSQC.

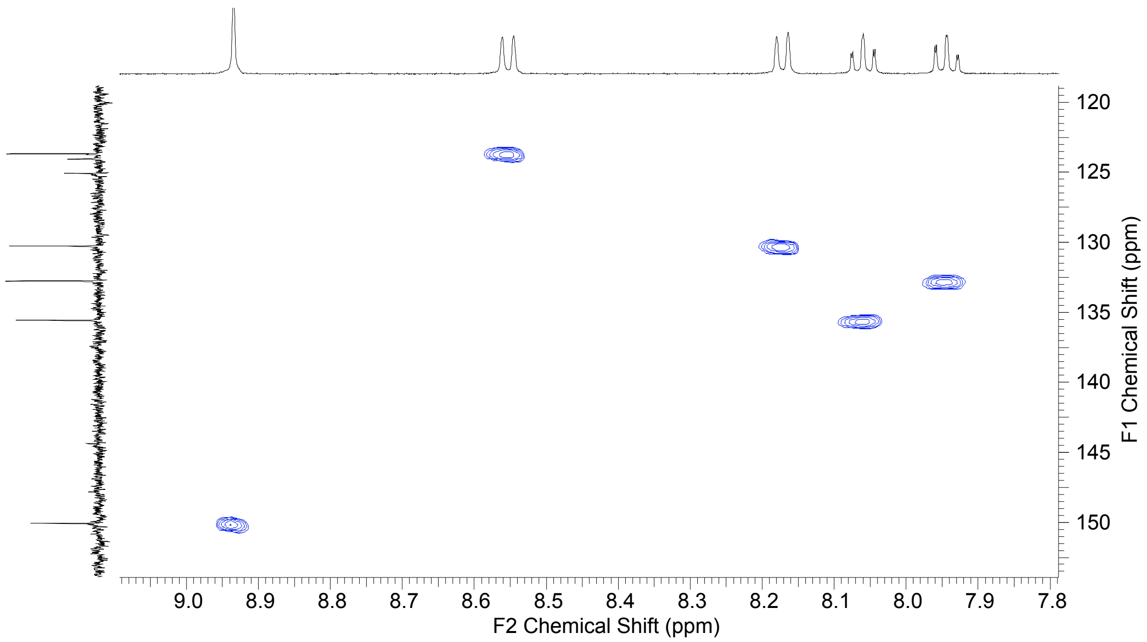

Figure S17. Compound **10** HSQC.

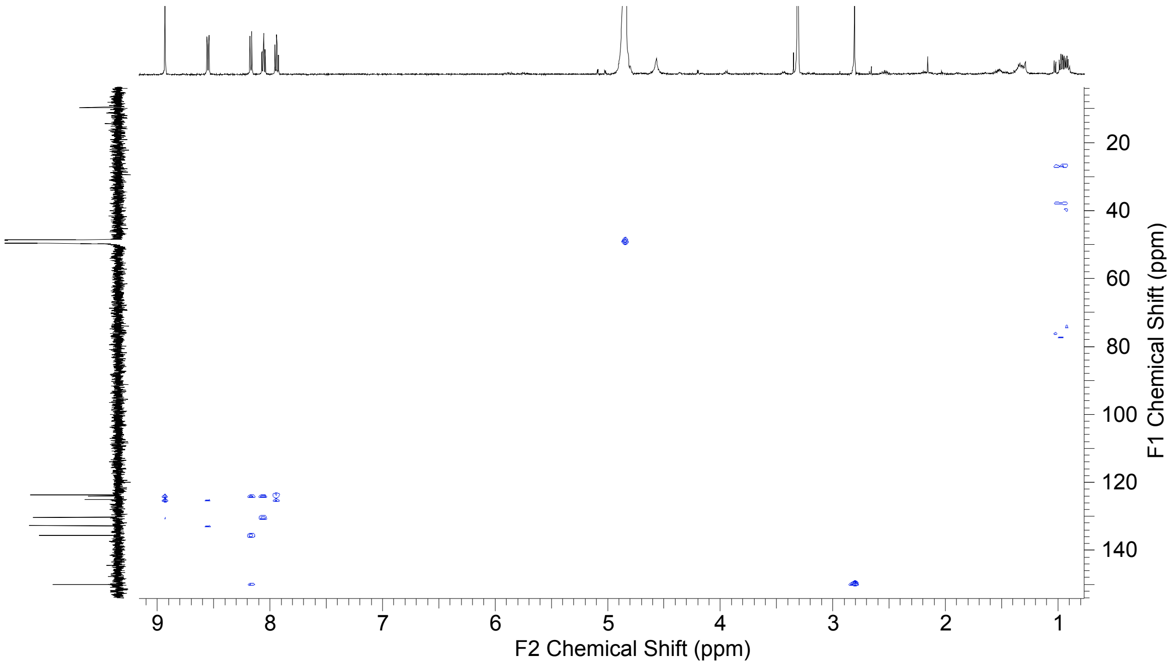

Figure S18. Compound **10** HMBC.

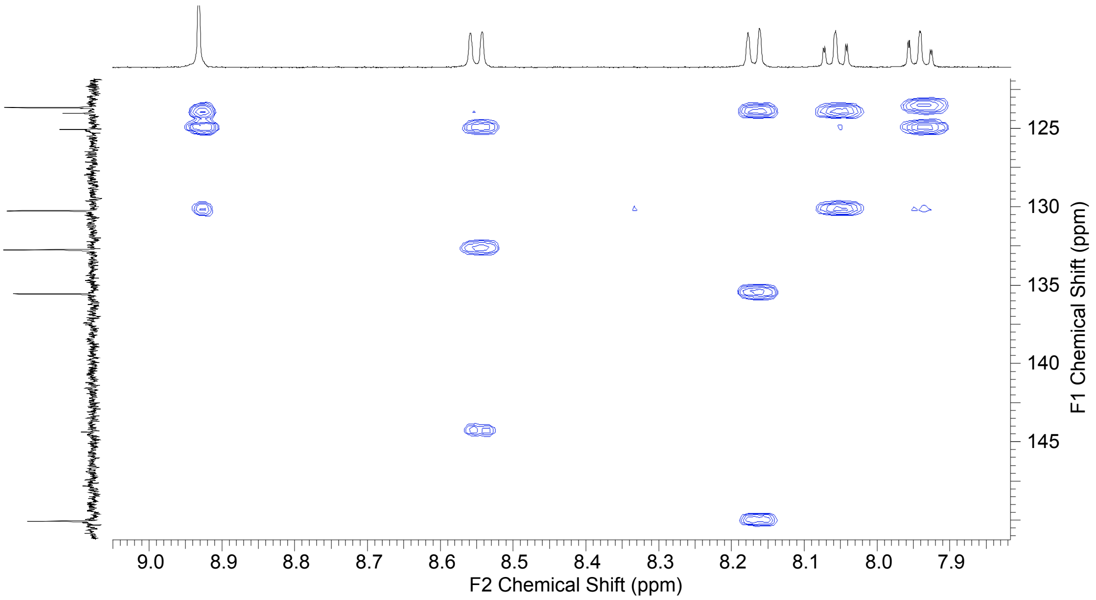

Figure S19. Compound **10** HMBC.

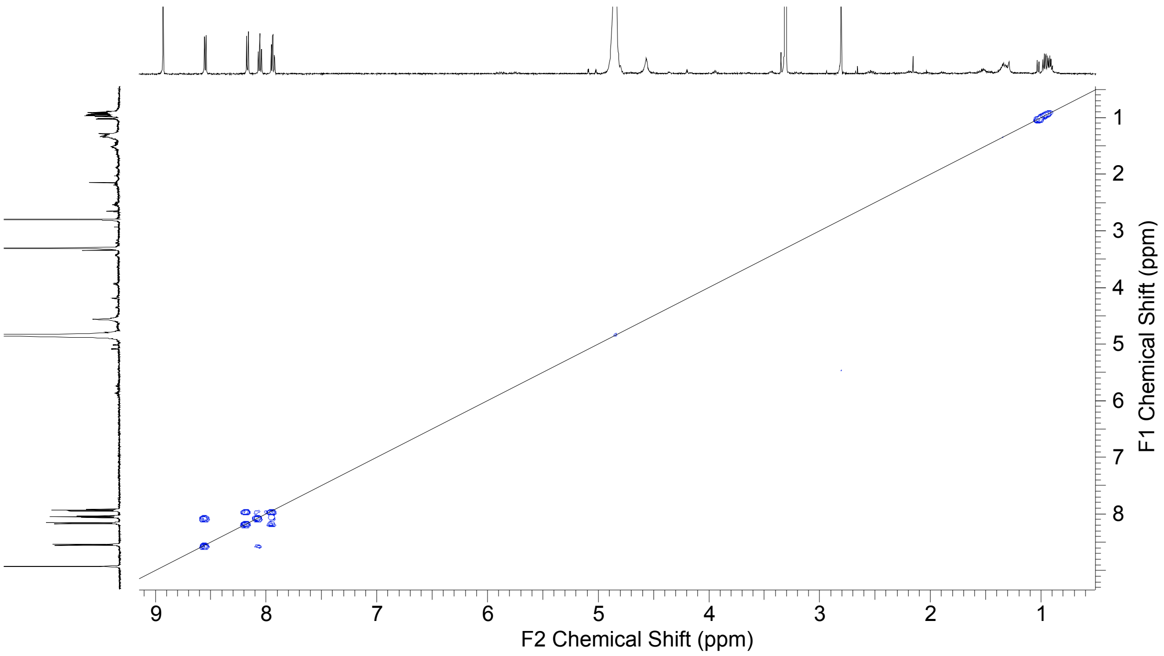

Figure S20. Compound **10** COSY.

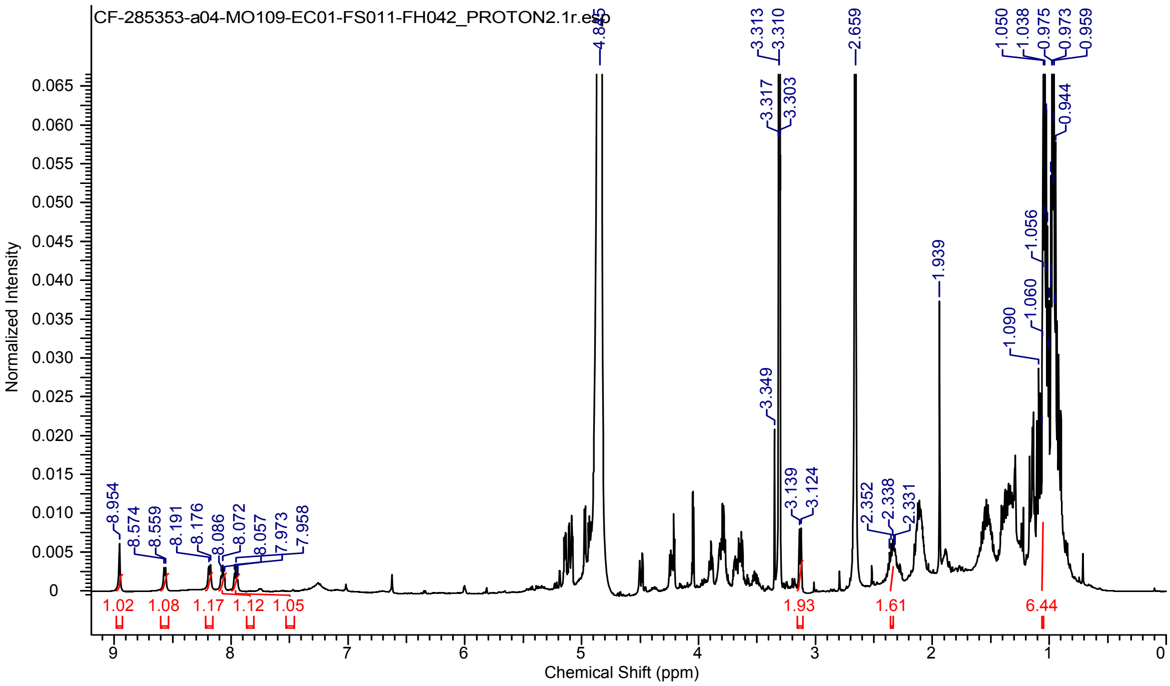

Figure S21. Mixture containing Compound **11** ^{1}H-RMN.

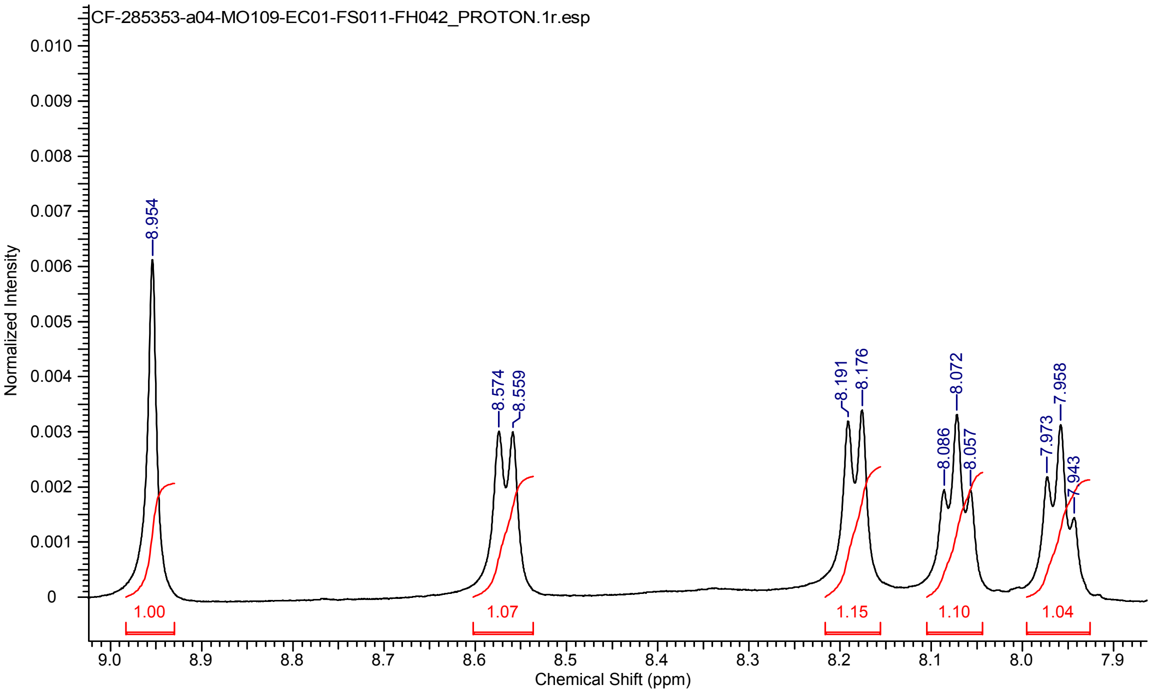

Figure S22. Compound **11** ^{1}H-RMN.

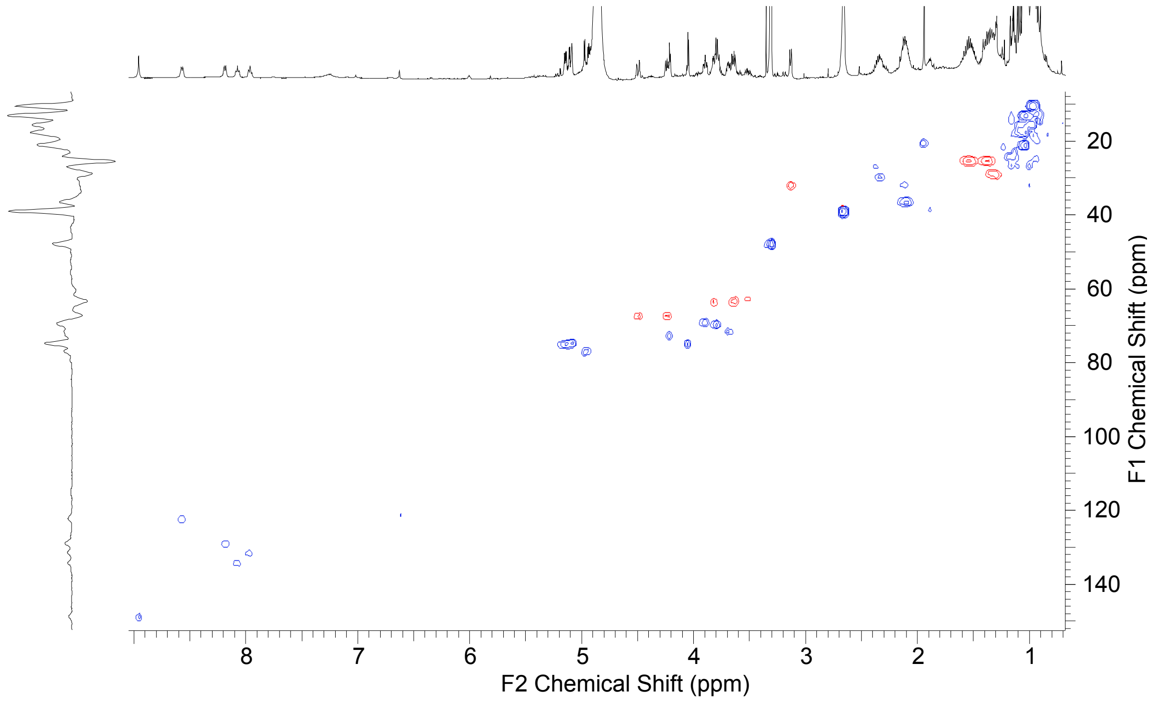

Figure S23. Compound **11** HSQC.

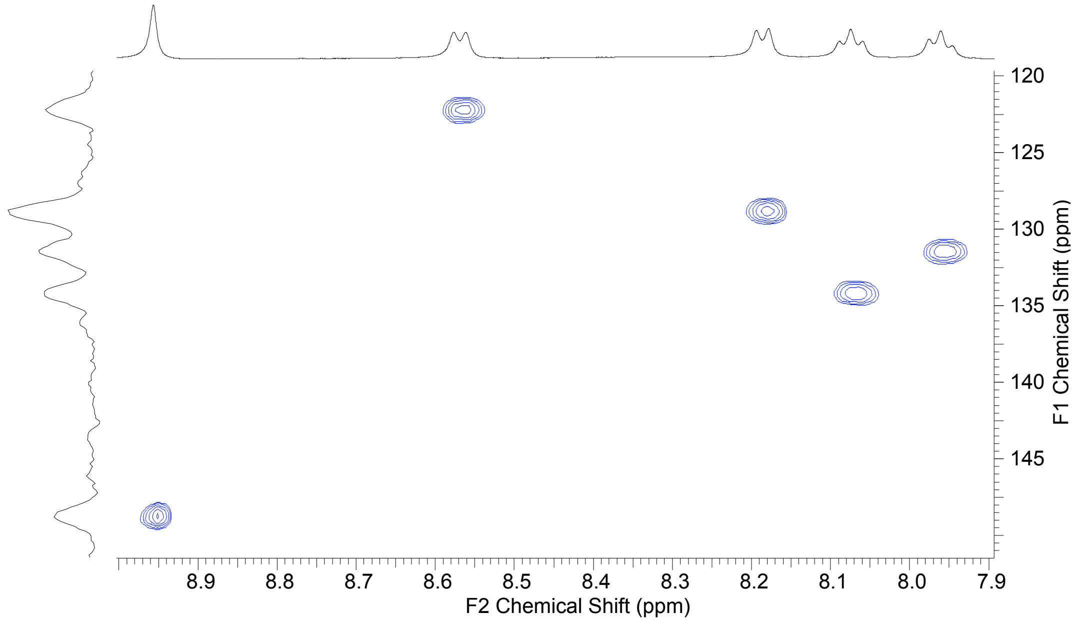

Figure S24. Compound **11** HSQC.

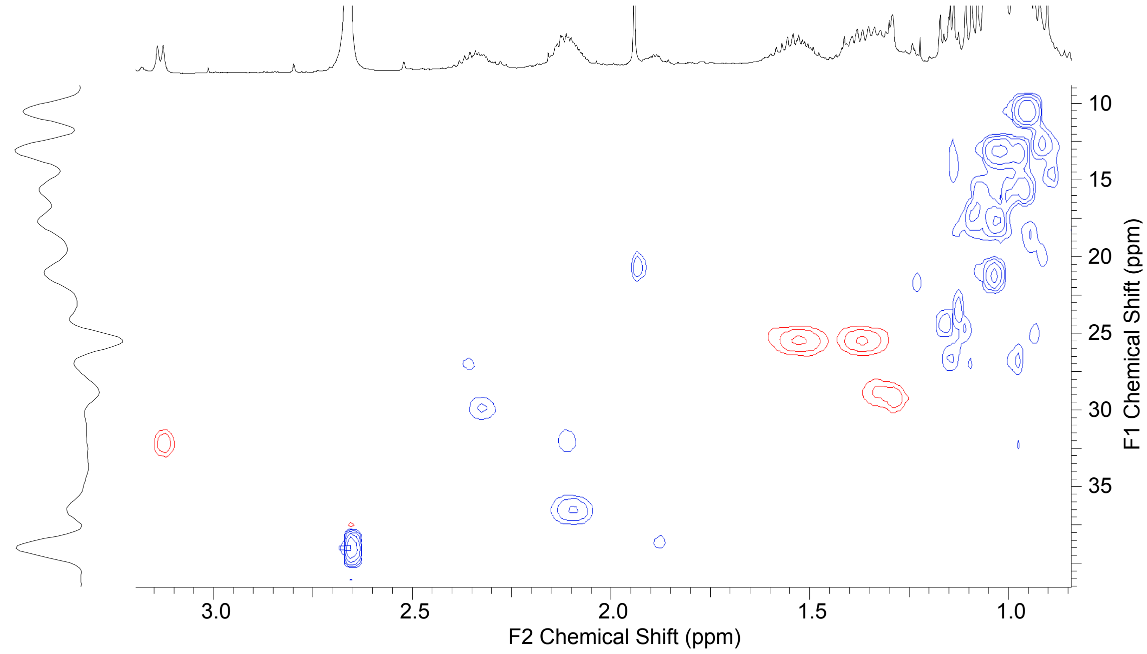

Figure S25. Compound **11** HSQC.

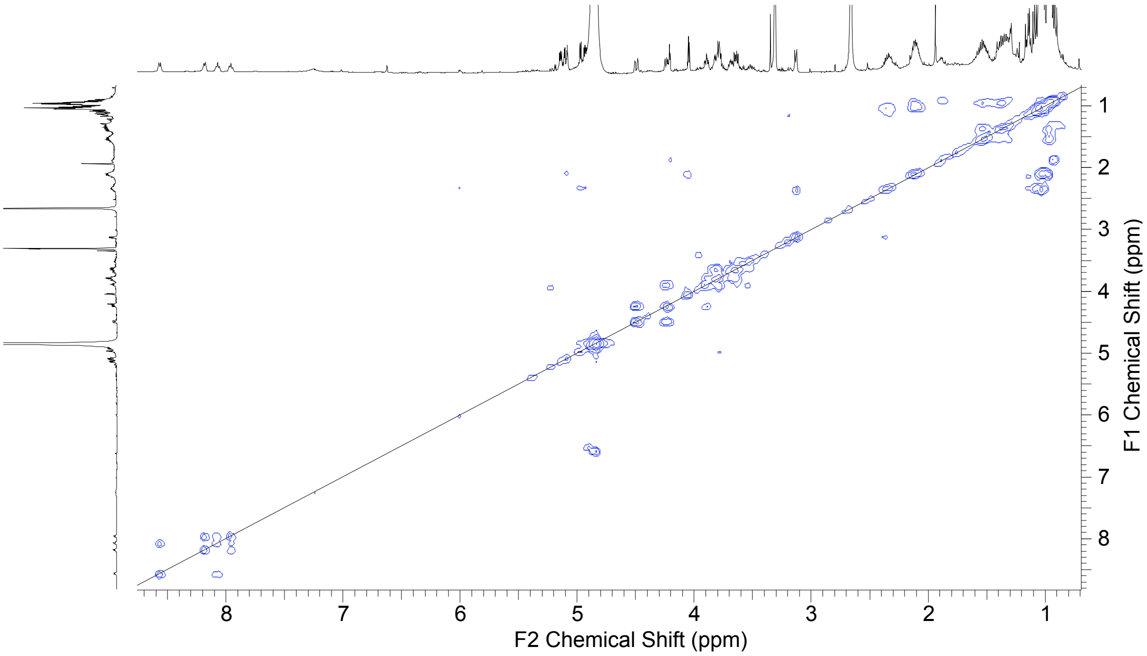

Figure S26. Compound **11** COSY.

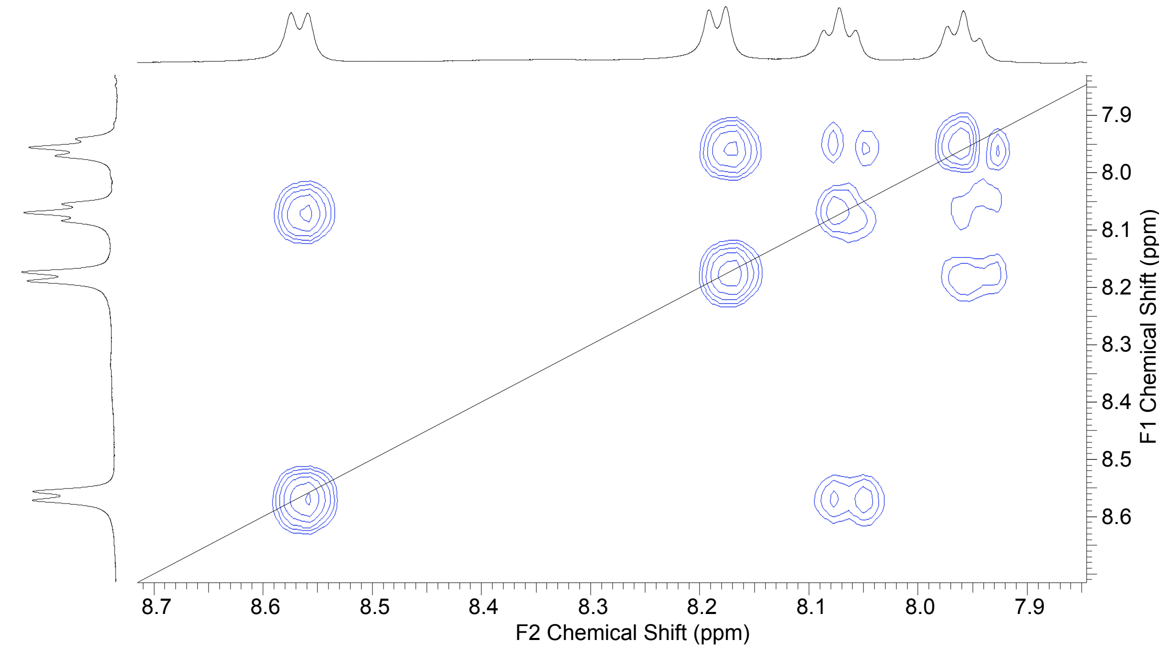

Figure S27. Compound **11** COSY.

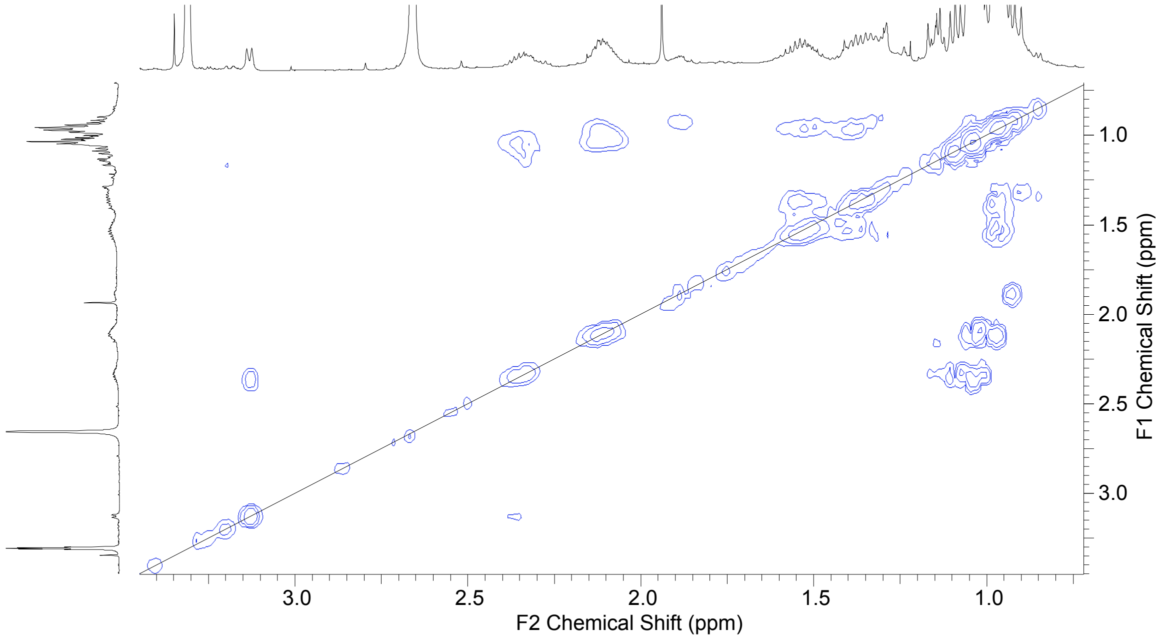

Figure S28. Compound **11** COSY.
